# An exposome-wide association study on body mass index in adolescents using the National Health and Nutrition Examination Survey (NHANES) 2003–2004 and 2013–2014 data

**DOI:** 10.1038/s41598-022-12459-z

**Published:** 2022-05-25

**Authors:** Nadine Haddad, Xanthi Andrianou, Christa Parrish, Stavros Oikonomou, Konstantinos C. Makris

**Affiliations:** Water and Health Laboratory, Cyprus International Institute for Environmental and Public Health, School of Health Sciences, Cyprus University of Technology, Irinis 95, 3041 Limassol, Cyprus

**Keywords:** Biomarkers, Risk factors

## Abstract

Excess weight is a public health challenge affecting millions worldwide, including younger age groups. The human exposome concept presents a novel opportunity to comprehensively characterize all non-genetic disease determinants at susceptible time windows. This study aimed to describe the association between multiple lifestyle and clinical exposures and body mass index (BMI) in adolescents using the exposome framework. We conducted an exposome-wide association (ExWAS) study using U.S. National Health and Nutrition Examination Survey (NHANES) 2003–2004 wave for discovery of associations between study population characteristics and zBMI, and used the 2013–2014 wave to replicate analysis. We included non-diabetic and non-pregnant adolescents aged 12–18 years. We performed univariable and multivariable linear regression analysis adjusted for age, sex, race/ethnicity, household smoking, and income to poverty ratio, and corrected for false-discovery rate (FDR). A total of 1899 and 1224 participants were eligible from 2003–2004 and 2013–2014 survey waves. Weighted proportions of overweight were 18.4% and 18.5% whereas those for obese were 18.1% and 20.6% in 2003–2004 and 2013–2014, respectively. Retained exposure agents included 75 laboratory (clinical and biomarkers of environmental chemical exposures) and 64 lifestyle (63 dietary and 1 physical activity) variables. After FDR correction, univariable regression identified 27 and 12 predictors in discovery and replication datasets, respectively, while multivariable regression identified 22 and 9 predictors in discovery and replication datasets, respectively. Six were significant in both datasets: alanine aminotransferase, gamma glutamyl transferase, segmented neutrophils number, triglycerides; uric acid and white blood cell count. In this ExWAS study using NHANES data, we described associations between zBMI, nutritional, clinical and environmental factors in adolescents. Future studies are warranted to investigate the role of the identified predictors as early-stage biomarkers of increased BMI and associated pathologies among adolescents and to replicate findings to other populations.

## Introduction

Overweight and obesity are described as an excessive adiposity or fat accumulation, often eliciting multiple health conditions leading to health impairments or even death^1–3^. Trends of overweight and obesity have been increasing over the past three decades, globally and across all age^4^. In a systematic analysis of global studies, the prevalence of overweight and obesity was estimated to have rose by 27.5% for adults and 47.1% for children and adolescents (2–19 years) between 1980 and 2013, hence, leading to an estimated increase in the number of overweight and obese individuals from 857 million to 2.1 billion during the same period^4^. In 2016, at least 340 million children and adolescents (5–19 years) were overweight or obese as per the World Health Organization (WHO) estimates^3^. In the United States alone, prevalence estimates of overweight and obesity among children and adolescents (2–19 years) also increased across race/ethnicities from 1966 to 1967 through 2017–2018, reaching 16% and 19%, respectively, or approximately 14.4 million in total^5^.

The physiological complications of overweight and obesity in children, adolescents and adults are well documented in the literature^6,7^. Adult onset of type 2 diabetes, hypertension, cardiovascular diseases, pulmonary, endocrine and gastrointestinal disorders as well as premature death were identified as major consequences of childhood and adult obesity^7,8^.

Obesity in adolescents tends to persist in adulthood. In a 12-year prospective study in Slovenia, more than half of 7-year-old overweight and obese children remained overweight or obese at the age of 18^9^. In a longitudinal follow-up of individuals enrolled in 1996 through 2007–2009 surveys of U.S. National Longitudinal Study of Adolescent Health, obese adolescents were 16 times more likely to develop severe adulthood obesity than normal or overweight adolescents^10^. Very high adolescent BMI (≥ 85th percentile) was associated with 30–40% higher adult mortality rate, as shown in a longitudinal assessment of national health surveys conducted in Norway between 1963 and 1999^11^.

Overweight and obesity are multifactorial with numerous identified risk factors. Genetic susceptibility is considered one of these risk factors, yet monogenic or polygenic forms of obesity are rare and complex interactions between genetic and environmental factors are biologically plausible^7,12,13^. While the genetic factors are measured in genome-wide association studies (GWAS), exposome studies are needed to capture all environmental factors complementary to the genome that influence health^14^. In the case of obesity and overweight, dietary behaviors (e.g., increased fat and calorie intake), lifestyle factors (e.g., physical inactivity and sedentary behavior), and psychological states (e.g., emotional stress and maladaptive coping strategies) are all considered possible risk factors of obesity onset and projection and their comprehensive examination is warranted for developing effective disease prevention programs^7^.

In an approach similar to GWAS, exposome-wide association studies (ExWAS) are often based on an agnostic, untargeted and hypothesis-generating approach aiming to identify associations between environmental factors and diseases outcomes^15–18^. The ExWAS approach was firstly used to explore the association of numerous nutrition and environmental factors with various health outcomes in adults, such as abdominal obesity^19^, blood pressure^20^, diabetes mellitus^15^ and telomere length^21^. The application of ExWAS in studying excess weight in younger age groups is still limited to a single study investigating the association between polycyclic aromatic hydrocarbons with childhood obesity among 6–17 years participants using data from the 1999–2016 National Health and Nutrition Examination Survey (NHANES) waves^22^.

Given the public health significance of the rising trends of overweight and obesity in younger age groups worldwide, comprehensive, data-driven and hypothesis-generating approaches are warranted to elucidate the role of environmental origins of obesity. Using data from the NHANES 2003–2004 and 2013–2014 waves, this study aimed to explore and describe the association between multiple lifestyle, clinical or other exposures and body mass index (BMI) of U.S. adolescents aged 12–18 years, using the exposome framework.

## Materials and methods

### Study population

We used the National Health and Nutrition Examination Survey (NHANES) data collected in the 2003–2004 and the 2013–2014 waves. We included participants between 12 and 18 years old at the time of the interview, who reported not being pregnant and not being told by a doctor or health professional they have diabetes, with non-missing BMI value. The selection of two surveys 10 years apart aimed to allow the description of exposome profile variations among adolescents in the United States.

The NHANES is a program of studies conducted every 2 years by the U.S. Center for Disease Control and Prevention (CDC) in a nationally representative multistage probability sample of non-institutionalized population living in the United States^23^. Each of the cross-sectional datasets includes demographic, dietary and health-related questions collected during the interview, in addition to laboratory tests, medical, dental and physiological measurements collected during the physical examination at the mobile examination centers^23^. All methods were performed in accordance with the relevant guidelines and regulations. The NCHS Ethics Review Board approved the survey protocols and written parental informed consent and child assent for participants ages 12–18 years were obtained for examination at the mobile examination center^24^.

### Study outcome

The study outcome was age- and sex-standardized body mass index (BMI) z-scores calculated using standard deviation scores from the U.S. CDC 2000 growth chart^25^. To describe weight status category, we used the 2010 CDC terminology for children overweight and obesity. The BMI z-score percentiles were used to classify children as being “underweight” (< 5th percentile); “healthy weight” (5th percentile < BMI z-score < 85th percentile); “overweight” (85th percentile ≤ BMI z-score ≤ 95th percentile); and “obese” (> 95th percentile)^26^.

### Selection of explanatory variables

**Figure 1:**
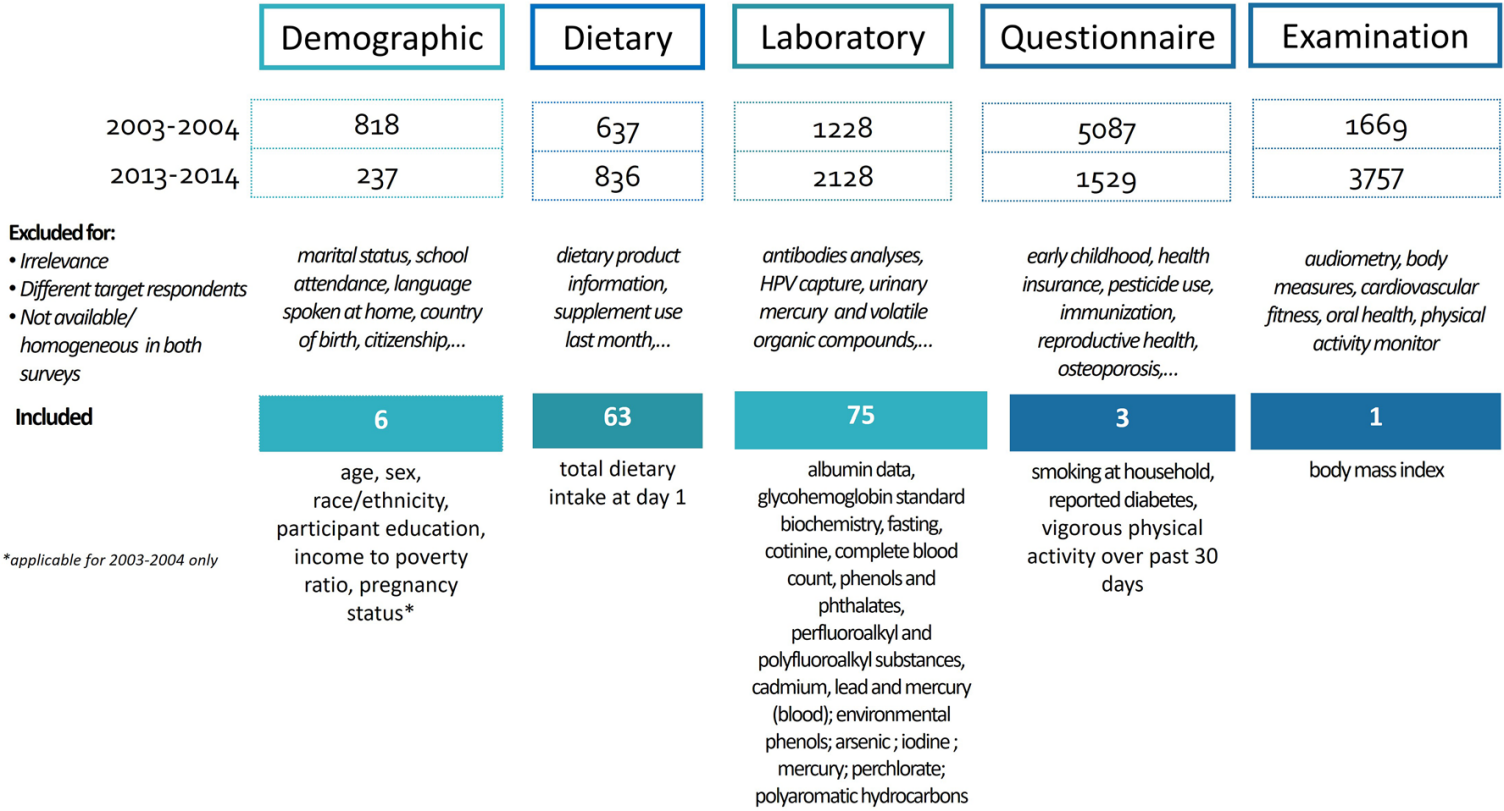
Initial selection of exposome variables from 2003–2004 and 2013–2014 NHANES waves.

We screened the data files from each of the five NHANES sections (i.e., demographics; dietary; examination; laboratory; and questionnaire data) of 2003–2004 and 2013–2014 surveys. In the initial screening, we selected variables: (i) available in both surveys with similar response format; (ii) targeting adolescents (12–18 years old); (iii) being relevant to the objective of the study (i.e., excluding: language spoken at home, audiometry examinations, dermatology, etc.); and (iv) for the laboratory variables, we included only parameters measured in individual samples (i.e., excluding brominated flame retardants (BFRs) that were measured in pooled samples). As a result, we retained 139 variables belonging to the following NHANES sections: laboratory data (75, i.e., clinical and biomarkers of environmental chemical exposures), dietary (63), and questionnaire (1) (Fig. 1 and Supplementary material).

Laboratory variables comprised the following measurements: serum and urinary albumin, serum cotinine, standard biochemistry, complete blood count and glycohemoglobin (n = 45); urinary measurements of phthalates (n = 9); perfluoroalkyl and polyfluoroalkyl substances (polyfluoroalkyl chemicals) (n = 7); urinary polyaromatic hydrocarbons (n = 6); blood cadmium, lead and mercury (n = 3); urinary environmental phenols (n = 1); urinary arsenic (n = 1); urinary iodine (n = 1); urinary mercury (n = 1); urinary perchlorate (n = 1). The 63 dietary variables referred to those collecting information about the dietary intake consumed during the 24-h period prior to the interview. The physical activity variable was retained from the questionnaire section.

### Variables transformation

All variables were evaluated as either continuous or categorical. For dietary and laboratory continuous variables, zero values were substituted with 1e^−10^; and all values were log-transformed, then scaled and centered. When necessary, categorical variables were recoded for a harmonized coding between the two survey datasets. For physical activity, a positive answer to vigorous physical activity “over past 30 days” (in 2003–2004 dataset) and “during a typical week” (in 2013–2014 dataset) were treated similarly. Particularly, participant’s educational level was categorized according to NHANES coding available in 2003–2004 demographic questionnaire: “Less than high school”, “High school including GED” and “More than high school”. We kept the grouping of race/ethnicity as included in NHANES questionnaire: Mexican American, Non-Hispanic White, Non-Hispanic Black, other Hispanic and other ethnicities. Age and poverty income ratio (PIR)—a ratio of family income to poverty threshold—was treated as a continuous variable. Household smoking was coded as “Yes/No”; with an answer of 1 or more smoking household members recoded as “Yes” in the 2013–2014 dataset to match the variable assessment in the 2003–2004 dataset.

### Statistical analysis

To account for the complex survey design, non-response and post-stratification, survey-weighted analysis was conducted using the appropriate sample weight as per NHANES analytical guidelines^27^. For each survey dataset, we created survey design datasets comprising the variables retained from each section along with their assigned sample weight: the demographic variables with the interview weight (wtint2yr); the laboratory variables and the physical activity with the Mobile Examination Center (MEC) exam weight (wtmec2yr); and the dietary variables with the dietary day 1 sample weight (wtdrd1). Specifically, for 2003–2004 dataset, polyfluoroalkyl, arsenic and mercury  variables using sub-sample A weight (wtsa2yr); phthalates and polyaromatic hydrocarbons variables using sub-sample B weight (wtsb2yr); environmental phenols, iodine and perchlorate variables using sub-sample C weight (wtsc2yr). As for 2013–2014 dataset, sub-sample A weight was used for arsenic, iodine, urine mercury, polyaromatic hydrocarbons and perchlorate variables; sub-sample B weight was used for environmental phenols, polyfluoroalkyl chemicals and phthalates data. For cadmium, lead and blood mercury variables, the MEC exam weight (wtmec2yr) and blood metal weight (wtsh2yr) were used for 2003–2004 and 2013–2014 datasets, respectively. For correlation between laboratory (standard biochemistry) and dietary explanatory variables, we applied the least common denominator approach and hence we used the weights of the dietary variables.

We considered the 2003–2004 dataset for discovery of associations between study population characteristics and zBMI, and replicated the analysis on the 2013–2014 dataset. Descriptive statistics and correlations between all explanatory variables were calculated separately for the discovery and replication datasets. Survey-weighted regressions were conducted to explore the associations between each of the explanatory variables and BMI z-score (univariable) and after adjusting for age, sex, race/ethnicity, household smoking and income to poverty ratio (multivariable). We applied the Benjamini–Hochberg method^28^ to generate false discovery rate (FDR) adjusted p-values separately for the univariable and multivariable analyses per dataset (FDR correction). Predictors with FDR adjusted p-value < 0.05 were considered significant. Next, we identified predictors that were commonly significant in the multivariable analysis of both the discovery and replication datasets.

To evaluate the possible interaction between sex, the statistically significant predictors retained in the previous step and zBMI, we repeated the multivariable analysis by adding the interaction term, separately for the discovery and replication datasets.

Additionally, a sensitivity analysis was conducted by excluding adolescents classified as obese and repeating univariable and multivariable regression on each of 2003–2004 and 2013–2014 datasets. All of the analysis was conducted using R version 4.1.2^29^, and the R studio version 2022.02.1^30^. The scripts used in the analysis as well as the detailed output of the analysis are available in the Supplementary Information. All survey-weighted descriptive and explanatory analyses were conducted using R *survey* package^31–33^. Data, scripts and outputs are available here: 10.5281/zenodo.6558979.

### Ethics approval and consent to participate

The NCHS Ethics Review Board approved the survey protocols and informed consent was obtained for all subjects.

### Consent for publication

No individual data that consent for publication shall be acquired is present in this manuscript.

## Results

### Descriptive characteristics

**Figure 2:**
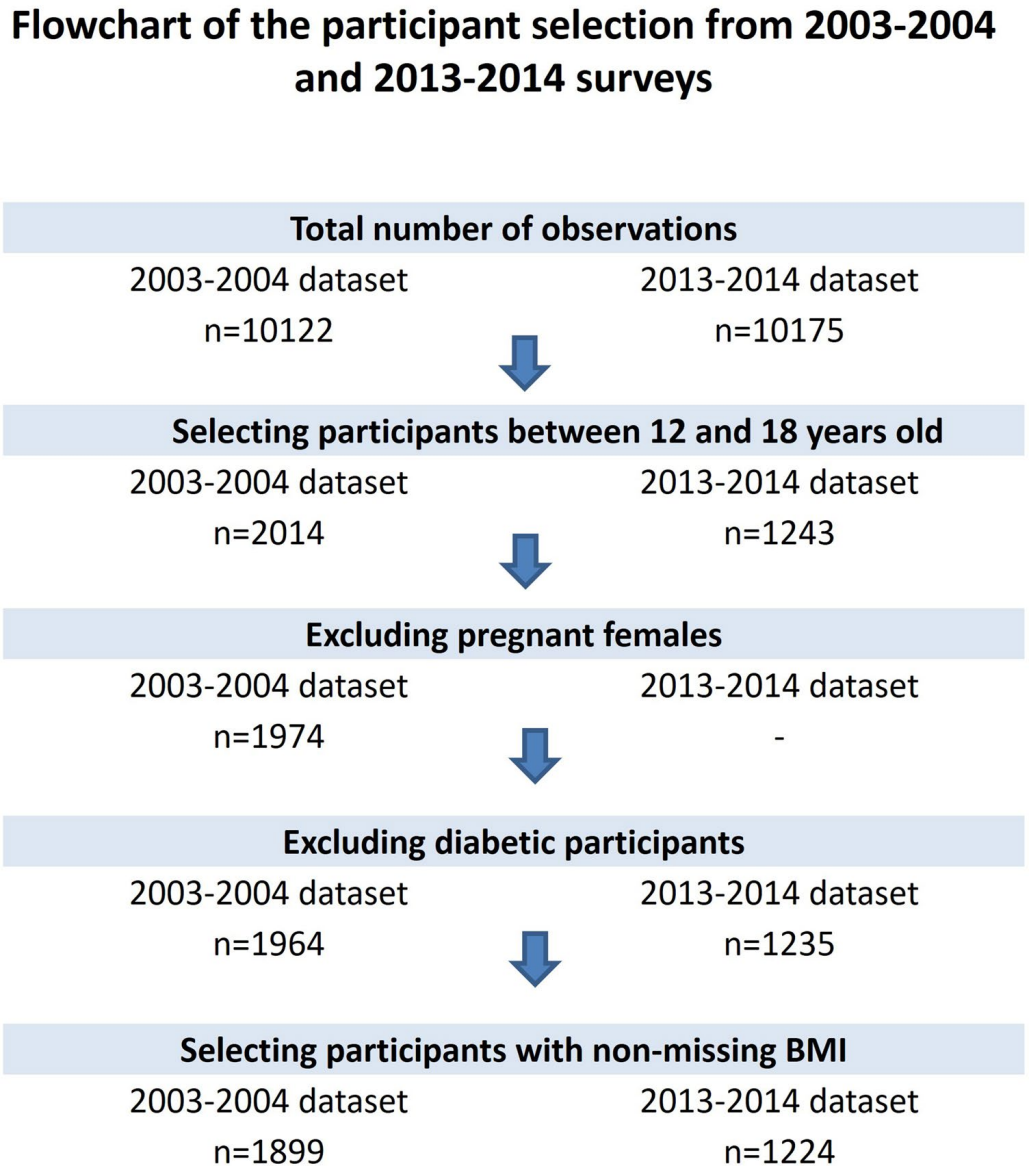
Flowchart of the participant selection from 2003–2004 and 2013–2014 surveys.

Following the selection criteria, 1899 and 1224 participants in total, were eligible from the 2003–2004 and 2013–2014 survey datasets, respectively (Fig. 2). The weighted mean age of adolescents was similar across the two, datasets with a mean of 15 (standard error (SE) 0.1) years of age. Similarly, almost half of our study population was males; non-Hispanic whites (64.5% and 54.2% in 2003–2004 and 2013–2014 survey datasets, respectively) having less than high school education (90.5% and 91.8% in 2003–2004 and 2013–2014 survey datasets, respectively). The mean poverty income ratio (PIR) was similar between the two surveys with a weighted mean of 2.6 (0.1 SE) and 2.4 (0.1 SE) in 2003–2004 and 2013–2014, respectively. The weighted proportion of reported household smoking in 2003–2004 was 25.1% (95% CI 19.9%, 30.4%) and 23.6% (95% CI 17.5%, 29.6%) in 2013–2014.

The weighted proportions of BMI categories were similar across the two surveys: “underweight” (2% in both survey waves); “healthy weight” (61.4% and 58.5% in 2003–2004 and 2013–2014, respectively); “overweight” (18% in both survey waves), and “obese” (18.1% and 20.6% in 2003–2004 and 2013–2014, respectively) (Table 1). No statistically significant (p > 0.05) differences were noted in the distribution of BMI categories between sexes in both survey waves (Supplementary Information S1).

**Table 1:** Estimated (weighted) background characteristics and categories of Body Mass Index of the study population from the 2003–2004 and 2013–2014 NHANES survey datasets.

	2003–2004	2013–2014
**Age [mean, (standard error)]**	15 (0.1)	15 (0.1)
**Sex (% [95% CI])**		
Male	50.7 (48.3%, 53.2%)	51.8 (47.9%, 55.7%)
Female	49.3 (46.8%, 51.7%)	48.2 (44.3%, 52.1%)
**Race/ethnicity (% [95% CI])**		
Mexican American	11 (5.4%, 16.5%)	15.4 (8.9%, 22%)
Non-Hispanic black	14.7 (9.7%, 19.8%)	14.3 (9.6%, 19%)
Non-Hispanic white	64.5 (54.8%, 74.2%)	54.2 (43.3%, 65%)
Other Hispanic	4.9 (2.3%, 7.4%)	6.9 (4.4%, 9.4%)
Other ethnicity	4.9 (2.9%, 6.8%)	9.2 (6.6%, 11.8%)
**Education level**		
Less than high school	90.5 (86.9%, 94.1%)	91.8 (89.3%, 94.3%)
More than high school	3.6 (1.9%, 5.2%)	1.6 (0.7%, 2.5%)
High school diploma including GED	5.9 (2.8%, 9%)	6.6 (4.4%, 8.8%)
**Poverty Income Ratio [mean, (standard error)]**	2.6 (0.1)	2.4 (0.1)
**Household smokers (% [95% CI])**		
Yes	25.1 (19.9%, 30.4%)	23.6 (17.5%, 29.6%)
Νο	74.9 (69.6%, 80.1%)	76.4 (70.4%, 82.5%)
**Body Mass Index (% [95% CI])**		
Underweight	2.2 (1.1%, 3.2%)	2.4 (1.1%, 3.8%)
Healthy weight	61.4 (55.7%, 67.1%)	58.5 (53.8%, 63.2%)
Overweight	18.4 (15.3%, 21.5%)	18.5 (15.5%, 21.5%)
Obese	18.1 (14.1%, 22.1%)	20.6 (16.2%, 24.9%)

### Exploratory ExWAS analysis using the 2003–2004 and 2013–2014 dataset

The univariate zBMI regression models for each of the 139 variables in the 2003–2004 dataset resulted in 54 predictors with a p-value < 0.05, of which only 27 were significant after FDR correction: 18 variables of laboratory variables (alanine aminotransferase (ALT); albumin; bicarbonate; total bilirubin; cholesterol; gamma glutamyl transferase (GGT); serum iron lactate dehydrogenase; lymphocyte number; mean cell hemoglobin; mean cell volume; platelet count; red blood cell count; segmented neutrophils number; sodium ; triglycerides; serum uric acid, and white blood cell count) and 9 dietary variables (folate as dietary folate equivalents; iron; lutein and zeaxanthin; riboflavin (vitamin B2); thiamin (vitamin B1); total folate; total sugars; vitamin B6, and retinol). All significant predictors (FDR adjusted p-value < 0.05) were negatively associated with zBMI, except for the following: ALT; cholesterol; GGT; lactate dehydrogenase; number of lymphocytes; platelet count; red blood cell count; segmented neutrophils number; triglycerides; serum uric acid, and white blood cell count (Supplementary Information S2).

Multivariable regression resulted in 45 predictors below the p-value cutoff of 0.05 of which only 12 significant after FDR correction: 11 laboratory variables (ALT; GGT; mean cell hemoglobin; mean cell volume; phosphorus; platelet count; red blood cell count; segmented neutrophils number; triglycerides; serum uric acid; white blood cell count) and 1 dietary variable (riboflavin (Vitamin B2)). Eight of these significant predictors were positively associated with BMI z-score (ALT; GGT); platelet count; red blood cell count; segmented neutrophils number; triglycerides; uric acid and white blood cell count) (Table 2).

**Table 2:** Variables significant in multivariable linear regressions adjusted for age, sex, race/ethnicity, household smoking and poverty income ratio in 2003–2004 and 2013–2014 survey datasets.

	2003–2004 discovery dataset			2013–2014 replication dataset		
	Estimate (S.E.)	p-value	FDR adjusted p-value	Estimate (S.E.)	p-value	FDR adjusted p-value
**Laboratory**						
Alanine aminotransferase (ALT,U/L)	0.383 (0.044)	< 0.001	0.008	0.405 (0.039)	< 0.001	0.002
Gamma glutamyl transferase (GGT, U/L)	0.341 (0.043)	< 0.001	0.010	0.416 (0.034)	< 0.001	0.002
Mean cell volume (fL)	− 0.174 (0.035)	0.003	0.040			
Segmented neutrophils number (1000 cell/μL)	0.211 (0.047)	0.004	0.048	0.273 (0.057)	0.003	0.047
Triglycerides (mmol/L)	0.285 (0.038)	< 0.001	0.010	0.355 (0.032)	0.001	0.002
Uric acid (μmol/L)	0.452 (0.046)	< 0.001	0.008	0.494 (0.066)	< 0.001	0.011
White blood cell count (1000 cells/μL)	0.188 (0.036)	0.002	0.040	0.269 (0.054)	0.002	0.043
Mean cell hemoglobin (pg)	− 0.163 (0.034)	0.003	0.045			
Phosphorus (mmol/L)	− 0.193 (0.042)	0.004	0.047			
Platelet count SI (1000 cells/μL)	0.17 (0.034)	0.002	0.040			
Red blood cell count (million cells/μL)	0.207 (0.038)	0.002	0.040			
Lactate dehydrogenase (U/L)				0.191 (0.035)	0.002	0.036
Monocyte number (1000 cells/μL)				0.215 (0.034)	0.001	0.028
**Nutrition (day 1)**						
Riboflavin (Vitamin B2) (mg)	− 0.17 (0.033)	0.002	0.040			
**Polyaromatic hydrocarbons (subsample A)**						
2-Hydroxynaphthalene (ng/L)				0.247 (0.047)	0.002	0.036

**Figure 3:**
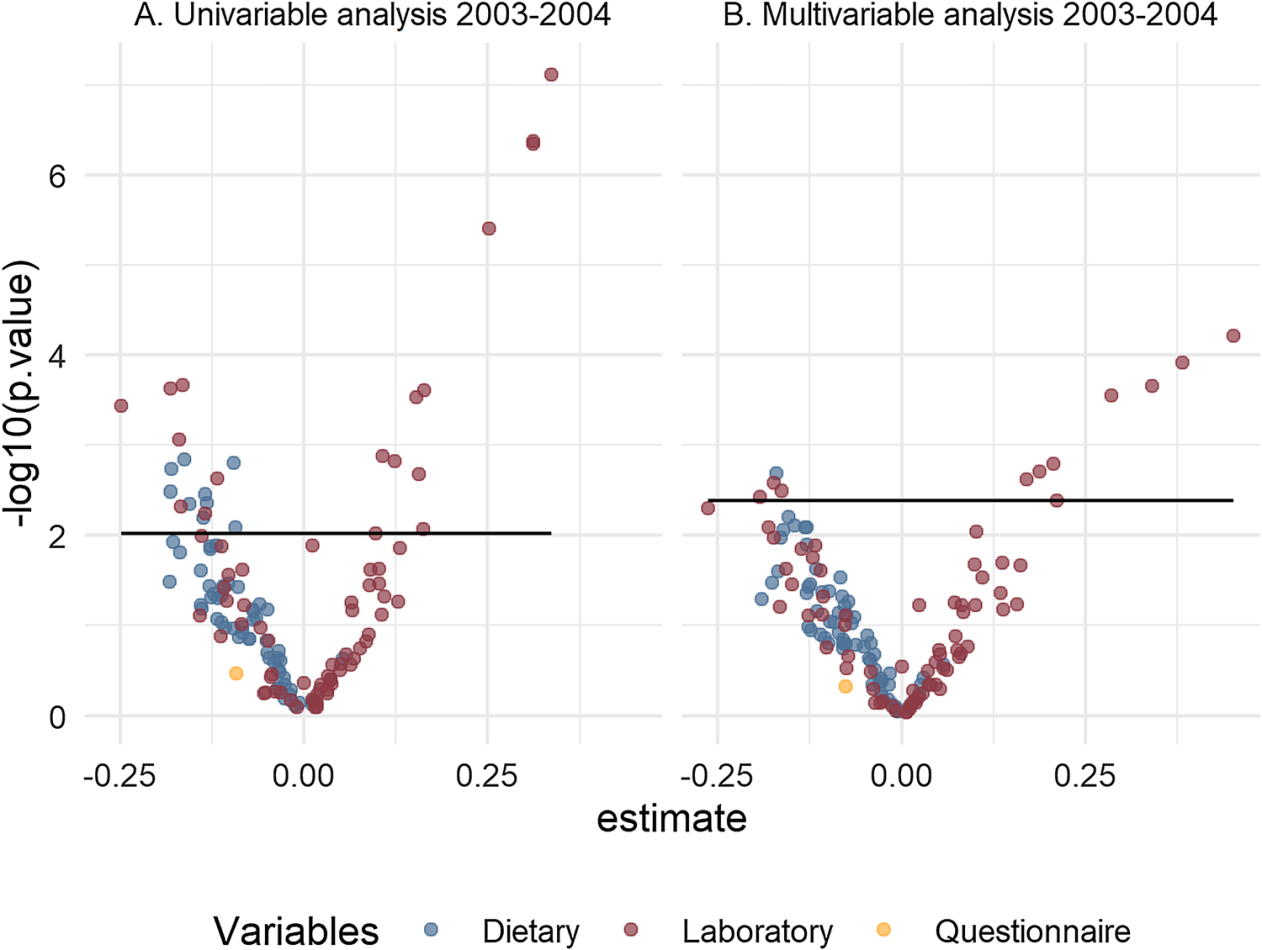
Volcano plots for the univariate and multivariable regressions in the 2003–2004 dataset.

**Figure4:**
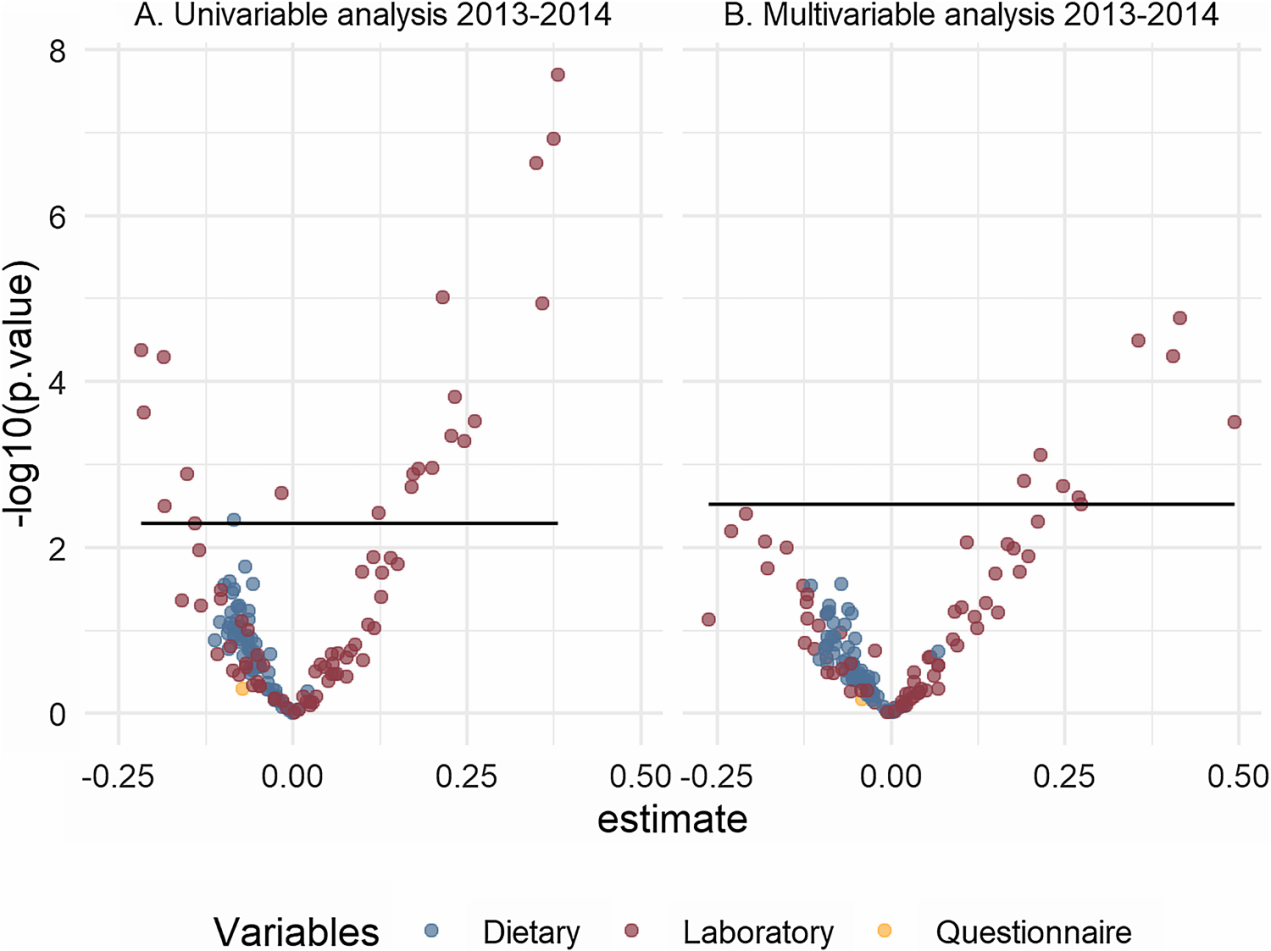
Volcano plots for the univariate regression and multivariable regressions in the 2013–2014 dataset.

For the replication dataset (2013–2014), the univariable regression models resulted in 39 predictors with a p-value < 0.05 of which only 22 were significant after FDR correction: 17 laboratory variables (ALT; albumin; GGT; globulin; serum iron; lactate dehydrogenase; lymphocyte number; mean cell hemoglobin; mean cell volume; monocyte number; platelet count; red cell distribution width; segmented neutrophils number; total bilirubin; triglycerides; serum uric acid; white blood cell count); 2 PAHs (1-hydroxyphenanthrene; 2-hydroxynaphthalene); 2 polyfluoroalkyl (perfluorodecanoic acid; perfluorobutane sulfonic acid) and 1 dietary variable (total sugars) (Supplementary Information S2). Multivariable regression resulted in 27 significant predictors of which only 9 laboratory variables were significant after FDR correction: 2-hydroxynaphthalene; ALT; GGT; lactate dehydrogenase; monocyte number; segmented neutrophils number; triglycerides; serum uric acid and white blood cell count (Table 2). All of these 9 variables had a positive significant association with zBMI. Volcano plots showing the distribution of the model estimates per predictor used in the ExWAS analysis are available in Figures 3 and 4.

**Figure 5:**
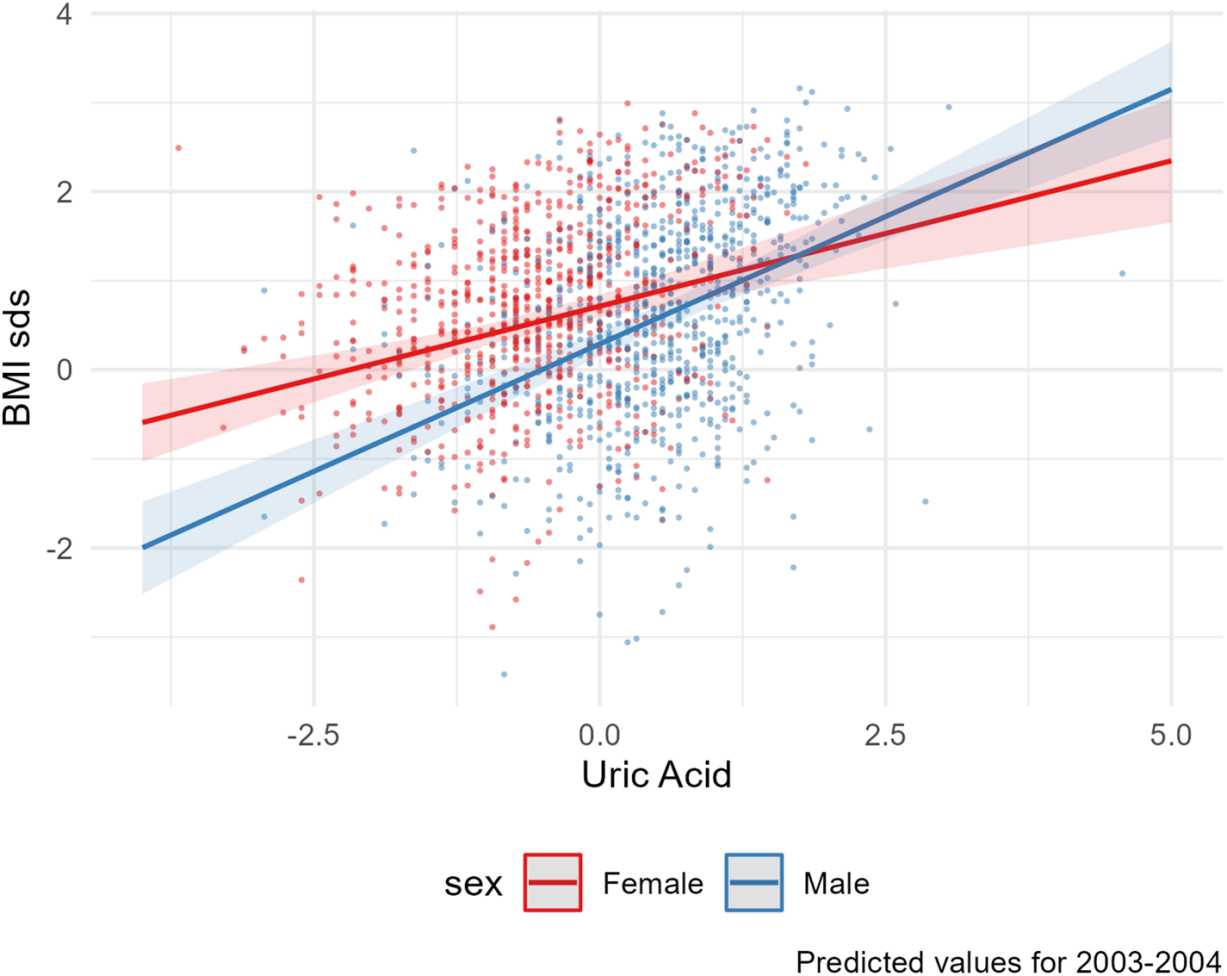
Scatter plot of the association between zBMI and uric acid in the 2003–2004 survey dataset: multivariable analysis including sex*uric acid as interaction term.

**Figure 6:**
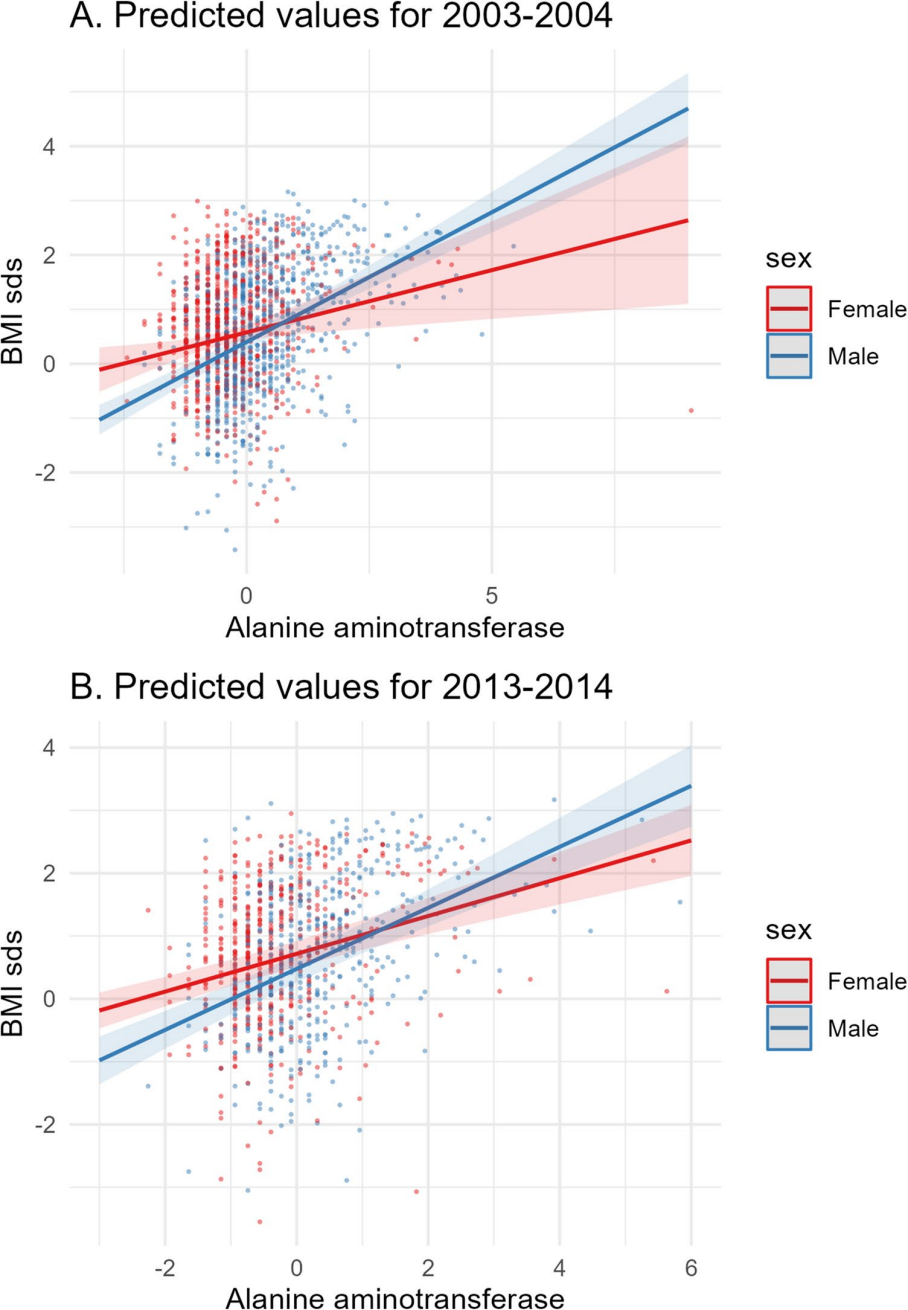
Scatter plots of the zBMI and alanine aminotransferase in the 2003–2004 and the 2013–2014 survey datasets including the line of the predicted values from the multivariable regression models that include the interaction term.

Six variables were commonly significant after FDR correction (FDR-adjusted p-values < 0.05) in multivariable analysis of both the discovery and replication datasets: ALT; GGT; segmented neutrophils number; triglycerides; serum uric acid and white blood cell count. In the second model for multivariable analysis that accounts for interaction between sex and each of the aforementioned predictors, the interaction term was significant for serum uric acid (estimate = 0.245, p = 0.022) and ALT (estimate = 0.248, p = 0.035) in the discovery dataset, and for ALT (estimate = 0.185, p = 0.03) only in the replication dataset (Figures 5 and 6). The full model estimates for variables with significant interaction terms are available in the Supplementary Information. Details on the regression analysis results can be found in the Supplementary Information.

### Sensitivity analysis

Sensitivity analysis was conducted on a sample of 1540 and 966 non-obese adolescents from the 2003–2004 and 2013–2014 survey datasets. None of the predictors were significant at univariable and multivariable analysis in 2003–2004 dataset. For 2013–2014 dataset, 6 laboratory variables (ALT; albumin; blood urea nitrogen; GGT; triglycerides and white blood cell count) and 3 dietary variables (cholesterol; folic acid and vitamin B12) were significant at the univariable analysis after FDR correction; however, none remain significant after adjusting for covariates in the multivariable analysis.

### Correlations

#### 2003–2004 dataset

When examining correlations between dietary and laboratory variables, weak coefficients were found overall; a maximum coefficient of r = 0.217 was noted for the correlation between selenium and blood urea nitrogen.

Looking specifically at the correlation between serum uric acid and both laboratory and dietary predictors; the correlation between uric acid and each of serum creatinine and hemoglobin showed the highest coefficients of 0.48 and 0.44, respectively, followed by a coefficient of 0.33 for the correlation between uric acid and GGT. On the other hand, the correlation between GGT and ALT had a coefficient of 0.52. A strong correlation was found between mean cell hemoglobin and mean cell volume (r = 0.94). A medium correlation was found for the following associations: albumin and total calcium (r = 0.56); albumin and total protein (r = 0.56); total bilirubin and iron (r = 0.43); triglycerides and total cholesterol (r = 0.35), while the association between GGT and platelet count was weak (r = 0.0265) (Supplementary Information S2).

#### 2013–2014 dataset

Similarly, weak coefficients were found when examining the correlation between dietary and laboratory variables, as noted in the correlation between selenium and each of protein (r = 0.9131); phosphorus (r = 0.8376) and blood urea nitrogen (r = 0.27). Medium correlations were noted for: GGT and ALT (r = 0.54); uric acid and each of creatinine (r = 0.45), hemoglobin (r = 0.39) and hematocrit (r = 0.39) (Supplementary Information S2).

## Discussion

We conducted an exposome-wide association study exploring 139 explanatory variables with respect to sex-specific BMI-for-age in non-diabetic and non-pregnant adolescents aged 12–18 years old in the 2003–2004 NHANES survey and used the 2013–2014 survey for validation. After adjusting for age, sex, race-ethnicity, household smoking, and poverty income ratio, we found ALT, GGT, segmented neutrophils number, triglycerides, serum uric acid and white blood cell count to have statistically significant associations with zBMI after adjusting for FDR in both the discovery and replication datasets, being in line with literature findings for adolescents.

Uric acid is the end product of purine metabolism in humans and its magnitude depends on dietary purines (from animal proteins, meat, seafood, beer and fructose sources), the degradation of endogenous purines as well as the renal and intestinal excretion of urate^34^. Although serum uric acid levels increase differently by sex from birth till adolescence, there is still no universally accepted threshold for defining hyperuricemia, or excess concentrations of serum uric acid in children and adolescents^35^. Elevated levels of uric acid have been associated with obesity and non-communicable diseases, such as kidney and cardiovascular diseases in children and adolescents^35,36^. Recent literature emphasized the association between uric acid and metabolic syndrome (MetS) outcomes in children and adolescents, such as glucose intolerance, central obesity, hypertension, and dyslipidemia^36–38^. Uric acid was associated with the prevalence of metabolic syndrome and its components, as shown in a cross-sectional analysis of 1370 adolescents (12–17 years of age) using data from NHANES 1999–2002; the unweighted prevalence of metabolic syndrome was ≈ 21% in the highest quartile (> 339 μmol/L) as compared to ≈ 10% in the third quartile (≤ 339 μmol/L), ≈ 4% in the second quartile (≤ 291 μmol/L) and < 1% among participants in the unweighted lowest quartile of serum concentrations of uric acid (≤ 250 μmol/L)^39^. A similar distribution of serum uric acid levels was observed in this study targeting non-diabetic adolescents for the 2003–2004 dataset (median: 297 μmol/L, interquartile range (IQR): [250 μmol/L, 351 μmol/L]) and 2013–2014 dataset (median: 297 μmol/L, IQR: [244 μmol/L, 351 μmol/L]). In the adjusted multivariable analysis, the strongest adjusted effect size of the association between various exposomic variables with zBMI was observed for uric acid in both discovery (estimate = 0.452) and replication (estimate = 0.707) datasets. After excluding non-obese participants, the association between zBMI and uric acid was no longer statistically significant, although it still showed the strongest effect size in both discovery and replication subsets (adjusted estimates of 0.24 and 0.39, respectively). Thus, our findings highlight the pathogenic role of elevated concentrations of uric acid in young obese age groups, as showcased in different studies. In a case–control study conducted in Italy among 120 children and adolescents with primary obesity (zBMI ≥ 97th percentile) and 50 healthy controls, carotid intima-media thickness was significantly correlated (r = 0.61; 95% CI 0.58–0.64) with the fourth quartile of uric acid among obese children regardless of the presence of metabolic syndrome, defined in the study as ≥ 3 or more of the following criteria: obesity, hypertension, low HDL cholesterol, elevated triglycerides, and impaired fasting glucose and/or insulin resistance^40^. On the other hand, the association between serum uric acid and cardiovascular diseases, irrespectively of BMI, has also been documented in a study conducted on an 1999–2006 unweighted NHANES sample of 12–17 years old adolescents; after adjusting for age, sex, race/ethnicity and BMI, the odds of having elevated blood pressure (mean systolic and/or diastolic blood pressure percentile ≥ 95th percentile) was 1.38 (95% CI 1.16–1.65) for each 0.1 mg/dL increase in uric acid^41^. In a randomized, double-blinded trial among pre-hypertensive obese adolescents (11–17 years old), patients treated with two mechanisms of urate reduction (allopurinol and probenecid) did not continue to gain weight during the 3-months study period and showed a similar and significant reduction in their systolic blood pressure by 10.2 mmHg and their diastolic by 9.0 mmHg in the two treatment groups as compared to the placebo group; thus, highlighting the role of uric acid as a biochemical mediator of increased blood pressure^42^.

The observed association of both uric acid and GGT with obesity and other cardiovascular risk factors has been previously documented in the literature. In a cross-sectional study of 2067 children and adolescents (6–20 years) in Hong Kong, a combined effect of the upper quartiles of both uric acid and GGT on obesity, low high-density lipoprotein cholesterol (HDL-C) levels and high blood pressure (adjusted odds ratios ranged from 1.63 to 5.82, all p < 0.005) was documented^37^. GGT, a liver enzyme implicated in the degradation of glutathione is associated with BMI, total cholesterol, diabetes mellitus (all components of MetS) as well as cardiovascular disease and all-cause mortality in adults^43^. The correlation between GGT and MetS and hypertension among younger age groups was demonstrated in a 10-year longitudinal study in Taiwan, where subjects (10–15 years) with higher baseline levels of GGT were at least twice more likely to develop MetS and hypertension during the follow-up period^44^. In our analysis, we showed a positive correlation, albeit weak, between uric acid and GGT in both discovery and replication datasets, without adjustment for zBMI.

Also, ALT had a statistically significant association with zBMI in the multivariable analysis using both the discovery and replication datasets. ALT is a liver enzyme related to fat liver accumulation and considered a useful biomarker for non-alcoholic fatty liver disease (NAFLD)^45^. The association between serum ALT and zBMI was previously documented in a study among adolescents (12–18 years) from NHANES III (1988–1994), in which overweight and obese study subjects were three to six times more likely to have higher levels of ALT (> 30 U/L) as compared to those with normal weight^46^. Similarly, a significant correlation between ALT, zBMI and metabolic syndrome was found among 5411 adolescents aged 12–19 years from NHANES 1999–2014; yet, with no significance increase in the prevalence of increased ALT over time^47^. On the other hand, elevated serum ALT levels (> 40 U/L) were also associated with markers of metabolic syndrome, as demonstrated in a study among adolescents 10–19 years old from the Korean National Health and Nutrition Examination Survey in 1998^48^.

Elevated triglycerides or hypertriglyceridemia is common among obese children and adolescents, and this component of metabolic syndrome is a known biomarker of cardiovascular disease risk^49^. In our analysis, a positive association between triglycerides and zBMI was found in each of the discovery (estimate = 0.285) and replication datasets (estimate = 0.444), but not in the sensitivity analysis in non-obese adolescents. This positive association found in our ExWAS study between triglycerides and zBMI is in line with the findings of a study on abnormal lipid levels among adolescents (12–19 years) in NHANES 1999–2006, in which 22% of overweight and 43% of obese had at least one abnormal lipid level including elevated triglycerides, the most common lipid abnormality associated with excess weight^50^.

Our analysis also showed the association between zBMI and inflammatory markers, such as white blood cell count and segmented neutrophils number. Such findings were also documented among adolescents^51–53^, suggesting that obesity-induced inflammation could start in childhood^54^.

None of the dietary variables remained significant at multivariable analysis in the discovery and replication datasets. Additionally, weak correlations were found between the laboratory and dietary variables in each of the discovery and replication datasets. On the other hand, none of the biomarkers of environmental chemical exposures remained significant after FDR correction at multivariable analysis in the discovery and replication datasets. Particularly, 2-hydroxynaphthalene was no longer significant at multivariable analysis in the replication dataset after FDR correction. Although the implicated biological mechanisms remain unclear, further studies are warranted to better understand the role of PAHs and other environmental pollutants in obesity pathogenesis.

The associations found between uric acid, GGT or ALT with zBMI using the whole study population were no longer statistically significant in the sensitivity analysis that included non-obese adolescents. This observation warrants for further investigation on the potential use of these biochemical parameters as biomarkers in the early stages of obesogenesis in adolescence or childhood. The sex specific trends observed in the association between the three aforementioned biochemical parameters and zBMI are also worth of detailed investigation in other population studies as they might be useful in future obesity screening and prevention programs in adolescence and/or earlier life stages.

The strength of this study lies in the agnostic nature of the ExWAS approach which allows for the simultaneous assessment of multiple parameters and their associations with different outcomes. The NHANES dataset is considered representative of the U.S. population; in effect, the weighted estimates of overweight and obesity prevalence in this U.S. study population (12–18 years old) were similar in the 2003–2004 (18.4% and 18.1%, respectively) and 2013–2014 survey datasets (18.5% and 20.6%, respectively). Moreover, the obesity prevalence estimates in both datasets were similar to the weighted estimates by the U.S. CDC of 17.4% (13.9–21.3%) for the 2003–2004 survey and 20.6% (16.2–25.6%) for the 2013–2014 survey^55^. The created models are considered as robust, being tested on two NHANES datasets, 10 years apart. Another strong feature of this ExWAS study was the inclusion of variables belonging to all exposome domains; the general external (individual household income), the specific external (dietary variables; education; household smoking and physical activity) and the internal domain (intrinsic and laboratory variables). Yet, in order to fully explore the exposome’s utility, it is encouraged to include additional groups of environmental components in relation to the studied health outcome^17^.

Due to the cross-sectional study design of NHANES, causal associations cannot be established and although the approach was as inclusive as possible, not all exposome parameters were available or could be included, e.g. chemical exposure data were not fully available in these NHANES surveys. On the other hand, the lack of observed associations between dietary factors and BMI might be interpreted by the use of a self-reported recall of the food items consumed during the past 24 hours; which might be subject to recall bias, under-reporting, over reporting, or omission of foods^56^. Additionally, our analysis did not account for the “dietary recall status” variable and hence subjects classified as not having a “reliable recall” were not excluded. In this study, we used only dietary intake measurements for 1 day as an adequate metric of describing population mean nutrient intake. Earlier studies have shown no significant differences between the 2 days’ dietary intake measurements available in NHANES for estimating population means^57,58^. However, multiple measures of daily dietary intake are recommended, particularly in exposome studies, to ensure sufficient capture of the within- and between- subject variability^59^. Another possible limitation is the use of only two surveys out of the total available NHANES year surveys; ExWAS studies integrating additional NHANES datasets as well as a bigger number of environmental exposure variables are warranted to improve our knowledge in the environmental determinants of obesogenesis. Moreover, we focused on the analysis of parameters such as dietary habits that are more directly linked with obesity and not in the analysis of external drivers of variability in these parameters such as mental health, food security, socioeconomic determinants. Although the poverty income ratio was used in the models as a proxy of socioeconomic status, more detailed exposome domain assessments including external parameters in the future will allow for the identification of effective population programs/interventions that tackle adolescent obesity.

Adolescence is a critical life window of susceptibility to metabolic diseases, such as, overweight and obesity during which ongoing children’s development may be perturbed by a suite of environmental stressors, including lifestyle/behavioral factors and dietary habits^58^. The methodological framework of the human exposome and its tools allow for a comprehensive assessment of multiple factors with respect to disease outcomes through an agnostic, untargeted and hypothesis-generating approach. The NHANES-based discovered and replicated predictors of zBMI among U.S. adolescents seem to be in line with the global literature and further highlight their importance as potential early-stage biomarkers of excess weight. Additional studies at younger age groups are warranted to better explore a broader suite of lifestyle, dietary and environmental determinants in association with these biomarkers of effect and their implications with metabolic disease process.

## Supplementary Information

Supplementary Information.

## Data Availability

The R code, scripts and output are made publicly available and they have been submitted on the Journal’s portal. There is supplementary visualization material based on these datasets, which can be easily customized to accommodate other NHANES surveys.

## Code availability

The code used for the analysis is available in the Supplementary Material. Data, scripts and outputs are available here: 10.5281/zenodo.6558979.
