# Supplementary material for: An exposome-wide association study on body mass index in adolescents using the National Health and Nutrition Examination Survey (NHANES) 2003–2004 and 2013–2014 data
Source: Sci Rep. 2022 May 25;12:8856. doi: 10.1038/s41598-022-12459-z (PMC9132896; doi:10.1038/s41598-022-12459-z)
Supplement: Supplementary file 1 — Supplementary Information. [file 41598_2022_12459_MOESM1_ESM.zip › SupplementaryMaterial_DataAnalysisDetails - Copy/wordoutput/DataDownload.docx]

Downloading US NHANES 2003-2004 and 2013-2014 data

Water and Health Laboratory - Cyprus University of Technology

03 February, 2022

# Downloading 2003-2004 data

theme_xpt <- c(
 # demographics
 "DEMO_C.XPT",
 # dietary
 "DR1TOT_C.XPT",
 # smoking data (household smoking)
 "SMQFAM_C.XPT",
 # diabetes data
 "DIQ_C.XPT",
 # Body Measurement data
 "BMX_C.XPT",
 # physical activity data
 "PAQ_C.XPT",
 ## Laboratory
 # Load 2003-2004 Albumin data
 "L16_C.XPT",
 # Standard Biochemistry data
 "L40_C.XPT",
 # Completele Blood Count data
 "L25_C.XPT",
 # Cotinine data
 "L06COT_C.XPT",
 "L10_C.XPT",
 # environmental phenols data
 "L24EPH_C.XPT",
 # Load phtalates urine data
 "L24PH_C.XPT",
 # Load arsenic data
 "L06UAS_C.XPT",
 #Load cadmium ,lead and mercury data
 "L06BMT_C.XPT",
 # Load iodine data
 "L06UIO_C.XPT",
 #Load mercury data
 "L06UHG_C.XPT",
 #Load perchlorate data
 "L04PER_C.XPT",
 # Load Polyaromatic Hydrocarbons (PAHs)
 "L31PAH_C.XPT",
 # Load Polyfluoroalkyl Chemicals data
 "L24PFC_C.XPT"
)

df_2003_2004 <- as.data.frame(theme_xpt) %>%
 mutate(complete_urls = paste0("https://wwwn.cdc.gov/Nchs/Nhanes/2003-2004/", theme_xpt)) %>%
 mutate(rds_name = paste0(str_replace_all(theme_xpt, "\\.XPT", "\\.rds"))) %>%
 mutate(htm_files = paste0("https://wwwn.cdc.gov/Nchs/Nhanes/2003-2004/", str_replace_all(theme_xpt, "\\.XPT", "\\.htm")))

xpt_files <- df_2003_2004$complete_urls

vars_summary_all <- data.frame()

ifelse(!dir.exists("rawdata/S2003_2004_rds/"),
 dir.create("rawdata/S2003_2004_rds/", recursive = TRUE), FALSE
)

## [1] FALSE

for (i in 1:length(xpt_files)) {
 tictoc::tic()
 data_url_string <- df_2003_2004[i, "complete_urls"]

 print("-----------------------------")
 print(paste0("Download: ", i))
 print(data_url_string)


 temp_data <- read_xpt(url(data_url_string)) %>%
 clean_names(case = "snake")

 saveRDS(temp_data, file = paste0("rawdata/S2003_2004_rds/", df_2003_2004[i, "rds_name"]))
 rm(temp_data, data_url_string)

 htm_url_string <- df_2003_2004[i, "htm_files"]
 print(paste0("Downloading from: :", htm_url_string))

 download.file(
 url = htm_url_string,
 destfile = paste0(
 "rawdata/S2003_2004_rds/",
 str_replace_all(htm_url_string, "https\\:\\/\\/wwwn\\.cdc.gov\\/Nchs\\/Nhanes\\/2003-2004\\/", "")
 )
 )


 vars_summary <- readHTMLList(readLines(htm_url_string), trim = T, header = T)[2] %>%
 as.data.frame() %>%
 separate(col = 1, sep = " - ", into = c("var_name", "summary")) %>%
 mutate(theme=theme_xpt[i]) ## adding information on the theme to use in renaming the variables in the next scripts

 vars_summary_all <- bind_rows(vars_summary_all, vars_summary)

 rm(htm_url_string, vars_summary)

 cat("\nMoving on to the next download")
 cat("\n-----------------------------\n")

 tictoc::toc()
}

## [1] "-----------------------------"
## [1] "Download: 1"
## [1] "https://wwwn.cdc.gov/Nchs/Nhanes/2003-2004/DEMO_C.XPT"
## [1] "Downloading from: :https://wwwn.cdc.gov/Nchs/Nhanes/2003-2004/DEMO_C.htm"

## Warning in readLines(htm_url_string): incomplete final line found on 'https://wwwn.cdc.gov/Nchs/
## Nhanes/2003-2004/DEMO_C.htm'

## Warning: Expected 2 pieces. Additional pieces discarded in 10 rows [6, 7, 8, 9, 10, 12, 15, 16,
## 17, 24].

##
## Moving on to the next download
## -----------------------------
## 16.52 sec elapsed
## [1] "-----------------------------"
## [1] "Download: 2"
## [1] "https://wwwn.cdc.gov/Nchs/Nhanes/2003-2004/DR1TOT_C.XPT"
## [1] "Downloading from: :https://wwwn.cdc.gov/Nchs/Nhanes/2003-2004/DR1TOT_C.htm"

## Warning in readLines(htm_url_string): incomplete final line found on 'https://wwwn.cdc.gov/Nchs/
## Nhanes/2003-2004/DR1TOT_C.htm'

##
## Moving on to the next download
## -----------------------------
## 50.2 sec elapsed
## [1] "-----------------------------"
## [1] "Download: 3"
## [1] "https://wwwn.cdc.gov/Nchs/Nhanes/2003-2004/SMQFAM_C.XPT"
## [1] "Downloading from: :https://wwwn.cdc.gov/Nchs/Nhanes/2003-2004/SMQFAM_C.htm"

## Warning in readLines(htm_url_string): incomplete final line found on 'https://wwwn.cdc.gov/Nchs/
## Nhanes/2003-2004/SMQFAM_C.htm'

##
## Moving on to the next download
## -----------------------------
## 3.74 sec elapsed
## [1] "-----------------------------"
## [1] "Download: 4"
## [1] "https://wwwn.cdc.gov/Nchs/Nhanes/2003-2004/DIQ_C.XPT"
## [1] "Downloading from: :https://wwwn.cdc.gov/Nchs/Nhanes/2003-2004/DIQ_C.htm"

## Warning in readLines(htm_url_string): incomplete final line found on 'https://wwwn.cdc.gov/Nchs/
## Nhanes/2003-2004/DIQ_C.htm'

## Warning: Expected 2 pieces. Additional pieces discarded in 1 rows [17].

##
## Moving on to the next download
## -----------------------------
## 7.5 sec elapsed
## [1] "-----------------------------"
## [1] "Download: 5"
## [1] "https://wwwn.cdc.gov/Nchs/Nhanes/2003-2004/BMX_C.XPT"
## [1] "Downloading from: :https://wwwn.cdc.gov/Nchs/Nhanes/2003-2004/BMX_C.htm"

## Warning in readLines(htm_url_string): incomplete final line found on 'https://wwwn.cdc.gov/Nchs/
## Nhanes/2003-2004/BMX_C.htm'

##
## Moving on to the next download
## -----------------------------
## 10.98 sec elapsed
## [1] "-----------------------------"
## [1] "Download: 6"
## [1] "https://wwwn.cdc.gov/Nchs/Nhanes/2003-2004/PAQ_C.XPT"
## [1] "Downloading from: :https://wwwn.cdc.gov/Nchs/Nhanes/2003-2004/PAQ_C.htm"

## Warning in readLines(htm_url_string): incomplete final line found on 'https://wwwn.cdc.gov/Nchs/
## Nhanes/2003-2004/PAQ_C.htm'

## Warning: Expected 2 pieces. Additional pieces discarded in 1 rows [14].

##
## Moving on to the next download
## -----------------------------
## 10.73 sec elapsed
## [1] "-----------------------------"
## [1] "Download: 7"
## [1] "https://wwwn.cdc.gov/Nchs/Nhanes/2003-2004/L16_C.XPT"
## [1] "Downloading from: :https://wwwn.cdc.gov/Nchs/Nhanes/2003-2004/L16_C.htm"

## Warning in readLines(htm_url_string): incomplete final line found on 'https://wwwn.cdc.gov/Nchs/
## Nhanes/2003-2004/L16_C.htm'

##
## Moving on to the next download
## -----------------------------
## 2.52 sec elapsed
## [1] "-----------------------------"
## [1] "Download: 8"
## [1] "https://wwwn.cdc.gov/Nchs/Nhanes/2003-2004/L40_C.XPT"
## [1] "Downloading from: :https://wwwn.cdc.gov/Nchs/Nhanes/2003-2004/L40_C.htm"

## Warning in readLines(htm_url_string): incomplete final line found on 'https://wwwn.cdc.gov/Nchs/
## Nhanes/2003-2004/L40_C.htm'

##
## Moving on to the next download
## -----------------------------
## 9.06 sec elapsed
## [1] "-----------------------------"
## [1] "Download: 9"
## [1] "https://wwwn.cdc.gov/Nchs/Nhanes/2003-2004/L25_C.XPT"
## [1] "Downloading from: :https://wwwn.cdc.gov/Nchs/Nhanes/2003-2004/L25_C.htm"

## Warning in readLines(htm_url_string): incomplete final line found on 'https://wwwn.cdc.gov/Nchs/
## Nhanes/2003-2004/L25_C.htm'

##
## Moving on to the next download
## -----------------------------
## 8.52 sec elapsed
## [1] "-----------------------------"
## [1] "Download: 10"
## [1] "https://wwwn.cdc.gov/Nchs/Nhanes/2003-2004/L06COT_C.XPT"
## [1] "Downloading from: :https://wwwn.cdc.gov/Nchs/Nhanes/2003-2004/L06COT_C.htm"

## Warning in readLines(htm_url_string): incomplete final line found on 'https://wwwn.cdc.gov/Nchs/
## Nhanes/2003-2004/L06COT_C.htm'

##
## Moving on to the next download
## -----------------------------
## 1.9 sec elapsed
## [1] "-----------------------------"
## [1] "Download: 11"
## [1] "https://wwwn.cdc.gov/Nchs/Nhanes/2003-2004/L10_C.XPT"
## [1] "Downloading from: :https://wwwn.cdc.gov/Nchs/Nhanes/2003-2004/L10_C.htm"

## Warning in readLines(htm_url_string): incomplete final line found on 'https://wwwn.cdc.gov/Nchs/
## Nhanes/2003-2004/L10_C.htm'

##
## Moving on to the next download
## -----------------------------
## 2.08 sec elapsed
## [1] "-----------------------------"
## [1] "Download: 12"
## [1] "https://wwwn.cdc.gov/Nchs/Nhanes/2003-2004/L24EPH_C.XPT"
## [1] "Downloading from: :https://wwwn.cdc.gov/Nchs/Nhanes/2003-2004/L24EPH_C.htm"

## Warning in readLines(htm_url_string): incomplete final line found on 'https://wwwn.cdc.gov/Nchs/
## Nhanes/2003-2004/L24EPH_C.htm'

##
## Moving on to the next download
## -----------------------------
## 2.77 sec elapsed
## [1] "-----------------------------"
## [1] "Download: 13"
## [1] "https://wwwn.cdc.gov/Nchs/Nhanes/2003-2004/L24PH_C.XPT"
## [1] "Downloading from: :https://wwwn.cdc.gov/Nchs/Nhanes/2003-2004/L24PH_C.htm"

## Warning in readLines(htm_url_string): incomplete final line found on 'https://wwwn.cdc.gov/Nchs/
## Nhanes/2003-2004/L24PH_C.htm'

##
## Moving on to the next download
## -----------------------------
## 5.89 sec elapsed
## [1] "-----------------------------"
## [1] "Download: 14"
## [1] "https://wwwn.cdc.gov/Nchs/Nhanes/2003-2004/L06UAS_C.XPT"
## [1] "Downloading from: :https://wwwn.cdc.gov/Nchs/Nhanes/2003-2004/L06UAS_C.htm"

## Warning in readLines(htm_url_string): incomplete final line found on 'https://wwwn.cdc.gov/Nchs/
## Nhanes/2003-2004/L06UAS_C.htm'

##
## Moving on to the next download
## -----------------------------
## 3.8 sec elapsed
## [1] "-----------------------------"
## [1] "Download: 15"
## [1] "https://wwwn.cdc.gov/Nchs/Nhanes/2003-2004/L06BMT_C.XPT"
## [1] "Downloading from: :https://wwwn.cdc.gov/Nchs/Nhanes/2003-2004/L06BMT_C.htm"

## Warning in readLines(htm_url_string): incomplete final line found on 'https://wwwn.cdc.gov/Nchs/
## Nhanes/2003-2004/L06BMT_C.htm'

##
## Moving on to the next download
## -----------------------------
## 4.78 sec elapsed
## [1] "-----------------------------"
## [1] "Download: 16"
## [1] "https://wwwn.cdc.gov/Nchs/Nhanes/2003-2004/L06UIO_C.XPT"
## [1] "Downloading from: :https://wwwn.cdc.gov/Nchs/Nhanes/2003-2004/L06UIO_C.htm"

## Warning in readLines(htm_url_string): incomplete final line found on 'https://wwwn.cdc.gov/Nchs/
## Nhanes/2003-2004/L06UIO_C.htm'

##
## Moving on to the next download
## -----------------------------
## 2.34 sec elapsed
## [1] "-----------------------------"
## [1] "Download: 17"
## [1] "https://wwwn.cdc.gov/Nchs/Nhanes/2003-2004/L06UHG_C.XPT"
## [1] "Downloading from: :https://wwwn.cdc.gov/Nchs/Nhanes/2003-2004/L06UHG_C.htm"

## Warning in readLines(htm_url_string): incomplete final line found on 'https://wwwn.cdc.gov/Nchs/
## Nhanes/2003-2004/L06UHG_C.htm'

##
## Moving on to the next download
## -----------------------------
## 2.03 sec elapsed
## [1] "-----------------------------"
## [1] "Download: 18"
## [1] "https://wwwn.cdc.gov/Nchs/Nhanes/2003-2004/L04PER_C.XPT"
## [1] "Downloading from: :https://wwwn.cdc.gov/Nchs/Nhanes/2003-2004/L04PER_C.htm"

## Warning in readLines(htm_url_string): incomplete final line found on 'https://wwwn.cdc.gov/Nchs/
## Nhanes/2003-2004/L04PER_C.htm'

##
## Moving on to the next download
## -----------------------------
## 2.33 sec elapsed
## [1] "-----------------------------"
## [1] "Download: 19"
## [1] "https://wwwn.cdc.gov/Nchs/Nhanes/2003-2004/L31PAH_C.XPT"
## [1] "Downloading from: :https://wwwn.cdc.gov/Nchs/Nhanes/2003-2004/L31PAH_C.htm"

## Warning in readLines(htm_url_string): incomplete final line found on 'https://wwwn.cdc.gov/Nchs/
## Nhanes/2003-2004/L31PAH_C.htm'

##
## Moving on to the next download
## -----------------------------
## 5.56 sec elapsed
## [1] "-----------------------------"
## [1] "Download: 20"
## [1] "https://wwwn.cdc.gov/Nchs/Nhanes/2003-2004/L24PFC_C.XPT"
## [1] "Downloading from: :https://wwwn.cdc.gov/Nchs/Nhanes/2003-2004/L24PFC_C.htm"

## Warning in readLines(htm_url_string): incomplete final line found on 'https://wwwn.cdc.gov/Nchs/
## Nhanes/2003-2004/L24PFC_C.htm'

##
## Moving on to the next download
## -----------------------------
## 5.27 sec elapsed

ifelse(!dir.exists("produceddata/"),
 dir.create("produceddata/", recursive = TRUE), FALSE
)

## [1] FALSE

saveRDS(vars_summary_all, "produceddata/vars_summary_all_2003v1.rds")

# Downloading 2013-2014 data

theme_xpt_1314 <- c(
 # demographics
 "DEMO_H.XPT",
 # dietary
 "DR1TOT_H.XPT",
 # smoking data (household somking)
 "SMQFAM_H.XPT",
 # diabetes data
 "DIQ_H.XPT",
 # Body Measurement data
 "BMX_H.XPT",
 # physical activity data
 "PAQ_H.XPT",
 ## Laboratory
 # Load 2013-2014 Albumin data
 "ALB_CR_H.XPT",
 # Standard Biochemistry data
 "BIOPRO_H.XPT",
 # Completele Blood Count data
 "CBC_H.XPT",
 # Cotinine data
 "COT_H.XPT",
 # glycohemoglobin data
 "GHB_H.XPT",
 # environmental phenols data
 "EPHPP_H.XPT",
 # Load phtalates urine data
 "PHTHTE_H.XPT",
 # Load arsenic data
 "UTAS_H.XPT",
 #Load cadmium and lead data
 "PBCD_H.XPT",
 # Load iodine data
 "UIO_H.XPT",
 #Load mercury data
 "UHG_H.XPT",
 #Load non-dioxin data
 "PCBPOL_H.XPT",
 #Load perchlorate data
 "PERNT_H.XPT",
 # Load Polyaromatic Hydrocarbons (PAHs)
 "PAH_H.XPT",
 # Load Polyfluoroalkyl Chemicals data
 "PFAS_H.XPT"
)

df_2013_2014 <- as.data.frame(theme_xpt_1314) %>%
 mutate(complete_urls = paste0("https://wwwn.cdc.gov/Nchs/Nhanes/2013-2014/", theme_xpt_1314)) %>%
 mutate(rds_name = paste0(str_replace_all(theme_xpt_1314, "\\.XPT", "\\.rds"))) %>%
 mutate(htm_files = paste0("https://wwwn.cdc.gov/Nchs/Nhanes/2013-2014/", str_replace_all(theme_xpt_1314, "\\.XPT", "\\.htm")))

xpt_files_1314 <- df_2013_2014$complete_urls

vars_summary_all_1314 <- data.frame()

ifelse(!dir.exists("rawdata/S2013_2014_rds/"),
 dir.create("rawdata/S2013_2014_rds/", recursive = TRUE), FALSE
)

## [1] FALSE

for (i in 1:length(xpt_files_1314)) {
 tictoc::tic()
 data_url_string <- df_2013_2014[i, "complete_urls"]

 print("-----------------------------")
 print(paste0("Download: ", i))
 print(data_url_string)


 temp_data <- read_xpt(url(data_url_string)) %>%
 clean_names(case = "snake")

 saveRDS(temp_data, file = paste0("rawdata/S2013_2014_rds/", df_2013_2014[i, "rds_name"]))
 rm(temp_data, data_url_string)

 htm_url_string <- df_2013_2014[i, "htm_files"]
 print(paste0("Downloading from: :", htm_url_string))

 download.file(
 url = htm_url_string,
 destfile = paste0(
 "rawdata/S2013_2014_rds/",
 str_replace_all(htm_url_string, "https\\:\\/\\/wwwn\\.cdc.gov\\/Nchs\\/Nhanes\\/2013-2014\\/", "")
 )
 )


 vars_summary <- readHTMLList(readLines(htm_url_string), trim = T, header = T)[2] %>%
 as.data.frame() %>%
 separate(col = 1, sep = " - ", into = c("var_name", "summary")) %>%
 mutate(theme=theme_xpt_1314[i])

 vars_summary_all_1314 <- bind_rows(vars_summary_all_1314, vars_summary)

 rm(htm_url_string, vars_summary)

 cat("\nMoving on to the next download")
 cat("\n-----------------------------\n")

 tictoc::toc()
}

## [1] "-----------------------------"
## [1] "Download: 1"
## [1] "https://wwwn.cdc.gov/Nchs/Nhanes/2013-2014/DEMO_H.XPT"
## [1] "Downloading from: :https://wwwn.cdc.gov/Nchs/Nhanes/2013-2014/DEMO_H.htm"

## Warning in readLines(htm_url_string): incomplete final line found on 'https://wwwn.cdc.gov/Nchs/
## Nhanes/2013-2014/DEMO_H.htm'

## Warning: Expected 2 pieces. Additional pieces discarded in 4 rows [6, 10, 16, 17].

##
## Moving on to the next download
## -----------------------------
## 20.03 sec elapsed
## [1] "-----------------------------"
## [1] "Download: 2"
## [1] "https://wwwn.cdc.gov/Nchs/Nhanes/2013-2014/DR1TOT_H.XPT"
## [1] "Downloading from: :https://wwwn.cdc.gov/Nchs/Nhanes/2013-2014/DR1TOT_H.htm"

## Warning in readLines(htm_url_string): incomplete final line found on 'https://wwwn.cdc.gov/Nchs/
## Nhanes/2013-2014/DR1TOT_H.htm'

##
## Moving on to the next download
## -----------------------------
## 132.54 sec elapsed
## [1] "-----------------------------"
## [1] "Download: 3"
## [1] "https://wwwn.cdc.gov/Nchs/Nhanes/2013-2014/SMQFAM_H.XPT"
## [1] "Downloading from: :https://wwwn.cdc.gov/Nchs/Nhanes/2013-2014/SMQFAM_H.htm"

## Warning in readLines(htm_url_string): incomplete final line found on 'https://wwwn.cdc.gov/Nchs/
## Nhanes/2013-2014/SMQFAM_H.htm'

##
## Moving on to the next download
## -----------------------------
## 4.77 sec elapsed
## [1] "-----------------------------"
## [1] "Download: 4"
## [1] "https://wwwn.cdc.gov/Nchs/Nhanes/2013-2014/DIQ_H.XPT"
## [1] "Downloading from: :https://wwwn.cdc.gov/Nchs/Nhanes/2013-2014/DIQ_H.htm"

## Warning in readLines(htm_url_string): incomplete final line found on 'https://wwwn.cdc.gov/Nchs/
## Nhanes/2013-2014/DIQ_H.htm'

##
## Moving on to the next download
## -----------------------------
## 70.48 sec elapsed
## [1] "-----------------------------"
## [1] "Download: 5"
## [1] "https://wwwn.cdc.gov/Nchs/Nhanes/2013-2014/BMX_H.XPT"
## [1] "Downloading from: :https://wwwn.cdc.gov/Nchs/Nhanes/2013-2014/BMX_H.htm"

## Warning in readLines(htm_url_string): incomplete final line found on 'https://wwwn.cdc.gov/Nchs/
## Nhanes/2013-2014/BMX_H.htm'

## Warning: Expected 2 pieces. Additional pieces discarded in 1 rows [12].

##
## Moving on to the next download
## -----------------------------
## 29.4 sec elapsed
## [1] "-----------------------------"
## [1] "Download: 6"
## [1] "https://wwwn.cdc.gov/Nchs/Nhanes/2013-2014/PAQ_H.XPT"
## [1] "Downloading from: :https://wwwn.cdc.gov/Nchs/Nhanes/2013-2014/PAQ_H.htm"
##
## Moving on to the next download
## -----------------------------
## 51.98 sec elapsed
## [1] "-----------------------------"
## [1] "Download: 7"
## [1] "https://wwwn.cdc.gov/Nchs/Nhanes/2013-2014/ALB_CR_H.XPT"
## [1] "Downloading from: :https://wwwn.cdc.gov/Nchs/Nhanes/2013-2014/ALB_CR_H.htm"
##
## Moving on to the next download
## -----------------------------
## 4.45 sec elapsed
## [1] "-----------------------------"
## [1] "Download: 8"
## [1] "https://wwwn.cdc.gov/Nchs/Nhanes/2013-2014/BIOPRO_H.XPT"
## [1] "Downloading from: :https://wwwn.cdc.gov/Nchs/Nhanes/2013-2014/BIOPRO_H.htm"

## Warning in readLines(htm_url_string): incomplete final line found on 'https://wwwn.cdc.gov/Nchs/
## Nhanes/2013-2014/BIOPRO_H.htm'

##
## Moving on to the next download
## -----------------------------
## 15.42 sec elapsed
## [1] "-----------------------------"
## [1] "Download: 9"
## [1] "https://wwwn.cdc.gov/Nchs/Nhanes/2013-2014/CBC_H.XPT"
## [1] "Downloading from: :https://wwwn.cdc.gov/Nchs/Nhanes/2013-2014/CBC_H.htm"

## Warning in readLines(htm_url_string): incomplete final line found on 'https://wwwn.cdc.gov/Nchs/
## Nhanes/2013-2014/CBC_H.htm'

##
## Moving on to the next download
## -----------------------------
## 11.69 sec elapsed
## [1] "-----------------------------"
## [1] "Download: 10"
## [1] "https://wwwn.cdc.gov/Nchs/Nhanes/2013-2014/COT_H.XPT"
## [1] "Downloading from: :https://wwwn.cdc.gov/Nchs/Nhanes/2013-2014/COT_H.htm"

## Warning in readLines(htm_url_string): incomplete final line found on 'https://wwwn.cdc.gov/Nchs/
## Nhanes/2013-2014/COT_H.htm'

##
## Moving on to the next download
## -----------------------------
## 7.61 sec elapsed
## [1] "-----------------------------"
## [1] "Download: 11"
## [1] "https://wwwn.cdc.gov/Nchs/Nhanes/2013-2014/GHB_H.XPT"
## [1] "Downloading from: :https://wwwn.cdc.gov/Nchs/Nhanes/2013-2014/GHB_H.htm"

## Warning in readLines(htm_url_string): incomplete final line found on 'https://wwwn.cdc.gov/Nchs/
## Nhanes/2013-2014/GHB_H.htm'

##
## Moving on to the next download
## -----------------------------
## 6.58 sec elapsed
## [1] "-----------------------------"
## [1] "Download: 12"
## [1] "https://wwwn.cdc.gov/Nchs/Nhanes/2013-2014/EPHPP_H.XPT"
## [1] "Downloading from: :https://wwwn.cdc.gov/Nchs/Nhanes/2013-2014/EPHPP_H.htm"

## Warning in readLines(htm_url_string): incomplete final line found on 'https://wwwn.cdc.gov/Nchs/
## Nhanes/2013-2014/EPHPP_H.htm'

##
## Moving on to the next download
## -----------------------------
## 17.06 sec elapsed
## [1] "-----------------------------"
## [1] "Download: 13"
## [1] "https://wwwn.cdc.gov/Nchs/Nhanes/2013-2014/PHTHTE_H.XPT"
## [1] "Downloading from: :https://wwwn.cdc.gov/Nchs/Nhanes/2013-2014/PHTHTE_H.htm"

## Warning in readLines(htm_url_string): incomplete final line found on 'https://wwwn.cdc.gov/Nchs/
## Nhanes/2013-2014/PHTHTE_H.htm'

##
## Moving on to the next download
## -----------------------------
## 12.36 sec elapsed
## [1] "-----------------------------"
## [1] "Download: 14"
## [1] "https://wwwn.cdc.gov/Nchs/Nhanes/2013-2014/UTAS_H.XPT"
## [1] "Downloading from: :https://wwwn.cdc.gov/Nchs/Nhanes/2013-2014/UTAS_H.htm"

## Warning in readLines(htm_url_string): incomplete final line found on 'https://wwwn.cdc.gov/Nchs/
## Nhanes/2013-2014/UTAS_H.htm'

##
## Moving on to the next download
## -----------------------------
## 2.61 sec elapsed
## [1] "-----------------------------"
## [1] "Download: 15"
## [1] "https://wwwn.cdc.gov/Nchs/Nhanes/2013-2014/PBCD_H.XPT"
## [1] "Downloading from: :https://wwwn.cdc.gov/Nchs/Nhanes/2013-2014/PBCD_H.htm"
##
## Moving on to the next download
## -----------------------------
## 7.34 sec elapsed
## [1] "-----------------------------"
## [1] "Download: 16"
## [1] "https://wwwn.cdc.gov/Nchs/Nhanes/2013-2014/UIO_H.XPT"
## [1] "Downloading from: :https://wwwn.cdc.gov/Nchs/Nhanes/2013-2014/UIO_H.htm"

## Warning in readLines(htm_url_string): incomplete final line found on 'https://wwwn.cdc.gov/Nchs/
## Nhanes/2013-2014/UIO_H.htm'

##
## Moving on to the next download
## -----------------------------
## 4.28 sec elapsed
## [1] "-----------------------------"
## [1] "Download: 17"
## [1] "https://wwwn.cdc.gov/Nchs/Nhanes/2013-2014/UHG_H.XPT"
## [1] "Downloading from: :https://wwwn.cdc.gov/Nchs/Nhanes/2013-2014/UHG_H.htm"

## Warning in readLines(htm_url_string): incomplete final line found on 'https://wwwn.cdc.gov/Nchs/
## Nhanes/2013-2014/UHG_H.htm'

##
## Moving on to the next download
## -----------------------------
## 6.89 sec elapsed
## [1] "-----------------------------"
## [1] "Download: 18"
## [1] "https://wwwn.cdc.gov/Nchs/Nhanes/2013-2014/PCBPOL_H.XPT"
## [1] "Downloading from: :https://wwwn.cdc.gov/Nchs/Nhanes/2013-2014/PCBPOL_H.htm"

## Warning in readLines(htm_url_string): incomplete final line found on 'https://wwwn.cdc.gov/Nchs/
## Nhanes/2013-2014/PCBPOL_H.htm'

##
## Moving on to the next download
## -----------------------------
## 18.44 sec elapsed
## [1] "-----------------------------"
## [1] "Download: 19"
## [1] "https://wwwn.cdc.gov/Nchs/Nhanes/2013-2014/PERNT_H.XPT"
## [1] "Downloading from: :https://wwwn.cdc.gov/Nchs/Nhanes/2013-2014/PERNT_H.htm"

## Warning in readLines(htm_url_string): incomplete final line found on 'https://wwwn.cdc.gov/Nchs/
## Nhanes/2013-2014/PERNT_H.htm'

##
## Moving on to the next download
## -----------------------------
## 3.7 sec elapsed
## [1] "-----------------------------"
## [1] "Download: 20"
## [1] "https://wwwn.cdc.gov/Nchs/Nhanes/2013-2014/PAH_H.XPT"
## [1] "Downloading from: :https://wwwn.cdc.gov/Nchs/Nhanes/2013-2014/PAH_H.htm"

## Warning in readLines(htm_url_string): incomplete final line found on 'https://wwwn.cdc.gov/Nchs/
## Nhanes/2013-2014/PAH_H.htm'

##
## Moving on to the next download
## -----------------------------
## 5.3 sec elapsed
## [1] "-----------------------------"
## [1] "Download: 21"
## [1] "https://wwwn.cdc.gov/Nchs/Nhanes/2013-2014/PFAS_H.XPT"
## [1] "Downloading from: :https://wwwn.cdc.gov/Nchs/Nhanes/2013-2014/PFAS_H.htm"

## Warning in readLines(htm_url_string): incomplete final line found on 'https://wwwn.cdc.gov/Nchs/
## Nhanes/2013-2014/PFAS_H.htm'

##
## Moving on to the next download
## -----------------------------
## 5.09 sec elapsed

ifelse(!dir.exists("produceddata/"),
 dir.create("produceddata/", recursive = TRUE), FALSE
)

## [1] FALSE

saveRDS(vars_summary_all_1314, "produceddata/vars_summary_all_2013v1.rds")

# Session information

sessionInfo()

## R version 4.1.2 (2021-11-01)
## Platform: x86_64-w64-mingw32/x64 (64-bit)
## Running under: Windows 10 x64 (build 19042)
##
## Matrix products: default
##
## locale:
## [1] LC_COLLATE=English_United Kingdom.1252 LC_CTYPE=English_United Kingdom.1252
## [3] LC_MONETARY=English_United Kingdom.1252 LC_NUMERIC=C
## [5] LC_TIME=English_United Kingdom.1252
## system code page: 65001
##
## attached base packages:
## [1] stats graphics grDevices utils datasets methods base
##
## other attached packages:
## [1] tictoc_1.0.1 XML_3.99-0.8 janitor_2.1.0 forcats_0.5.1 stringr_1.4.0
## [6] dplyr_1.0.7 purrr_0.3.4 readr_2.1.1 tidyr_1.1.4 tibble_3.1.6
## [11] ggplot2_3.3.5 tidyverse_1.3.1 haven_2.4.3
##
## loaded via a namespace (and not attached):
## [1] nlme_3.1-153 fs_1.5.2 lubridate_1.8.0 insight_0.15.0
## [5] progress_1.2.2 httr_1.4.2 inspectdf_0.0.11 tools_4.1.2
## [9] backports_1.4.1 utf8_1.2.2 R6_2.5.1 sjlabelled_1.1.8
## [13] DBI_1.1.2 colorspace_2.0-2 withr_2.4.3 tidyselect_1.1.1
## [17] prettyunits_1.1.1 emmeans_1.7.2 compiler_4.1.2 performance_0.8.0
## [21] cli_3.1.1 rvest_1.0.2 xml2_1.3.3 sandwich_3.0-1
## [25] bayestestR_0.11.5 scales_1.1.1 mvtnorm_1.1-3 digest_0.6.29
## [29] minqa_1.2.4 rmarkdown_2.11 pkgconfig_2.0.3 htmltools_0.5.2
## [33] lme4_1.1-27.1 dbplyr_2.1.1 fastmap_1.1.0 rlang_0.4.12
## [37] readxl_1.3.1 rstudioapi_0.13 generics_0.1.1 zoo_1.8-9
## [41] jsonlite_1.7.3 sjPlot_2.8.10 magrittr_2.0.2 parameters_0.16.0
## [45] Matrix_1.3-4 Rcpp_1.0.8 munsell_0.5.0 fansi_1.0.2
## [49] ggfittext_0.9.1 lifecycle_1.0.1 stringi_1.7.6 multcomp_1.4-18
## [53] yaml_2.2.2 snakecase_0.11.0 MASS_7.3-54 plyr_1.8.6
## [57] grid_4.1.2 sjmisc_2.8.9 crayon_1.4.2 lattice_0.20-45
## [61] ggeffects_1.1.1 splines_4.1.2 sjstats_0.18.1 hms_1.1.1
## [65] knitr_1.37 pillar_1.6.5 boot_1.3-28 estimability_1.3
## [69] effectsize_0.6.0.1 codetools_0.2-18 reprex_2.0.1 glue_1.6.1
## [73] evaluate_0.14 modelr_0.1.8 vctrs_0.3.8 nloptr_2.0.0
## [77] tzdb_0.2.0 cellranger_1.1.0 gtable_0.3.0 assertthat_0.2.1
## [81] datawizard_0.2.3 xfun_0.29 xtable_1.8-4 broom_0.7.12
## [85] coda_0.19-4 survival_3.2-13 TH.data_1.1-0 ellipsis_0.3.2
