# Supplementary material for: An exposome-wide association study on body mass index in adolescents using the National Health and Nutrition Examination Survey (NHANES) 2003–2004 and 2013–2014 data
Source: Sci Rep. 2022 May 25;12:8856. doi: 10.1038/s41598-022-12459-z (PMC9132896; doi:10.1038/s41598-022-12459-z)
Supplement: Supplementary file 1 — Supplementary Information. [file 41598_2022_12459_MOESM1_ESM.zip › SupplementaryMaterial_DataAnalysisDetails - Copy/wordoutput/S1_DataPrep.docx]

Environment-wide association study on body mass index of 12-18 year-olds, US NHANES 2003-2004 2003-2004 and 2013-2014

Water and Health Laboratory - Cyprus University of Technology

# Discovery: 2003-2004 NHANES data

Downloaded from: [***https://wwwn.cdc.gov/nchs/nhanes/ContinuousNhanes/Default.aspx?BeginYear=2003***](https://wwwn.cdc.gov/nchs/nhanes/ContinuousNhanes/Default.aspx?BeginYear=2003)

## Introduction - participant selection

- Total number of records/participants in the combined dataset: 10122

Exclusion:

- Records/participants <12 and >18 years-old: 8108
- Pregnant participants: 40
- Diabetics: 10
- Missing BMI: 65

# Replication: 2013-2014 NHANES data

Downloaded from: [***https://wwwn.cdc.gov/nchs/nhanes/ContinuousNhanes/Default.aspx?BeginYear=2013***](https://wwwn.cdc.gov/nchs/nhanes/ContinuousNhanes/Default.aspx?BeginYear=2013)

## Introduction - participant selection

- Total number of records/participants in the combined dataset: 10175

Exclusion:

- Records/participants <12 and >18 years-old: 8896
- Pregnant participants: # no filtering for this variable in 2013-2014
  - Diabetics: 8
- Missing BMI: 47

# Exclusion of variables based on % of missing values

# 2003-2004

## Descriptives - Background: unweighted statistics 2003-2004

Background participant characteristics: Descriptives of the categorical variables.

|  | Overall |
| --- | --- |
| n | 1899 |
| sex = Male (%) | 999 (52.6) |
| smoker_home = Yes (%) | 429 (22.8) |
| ethnicity (%) |  |
| Mexican American | 577 (30.4) |
| Non-Hispanic Black | 683 (36.0) |
| Non-Hispanic White | 514 (27.1) |
| Other | 67 ( 3.5) |
| Other Hispanic | 58 ( 3.1) |
| edu (%) |  |
| High school diploma including GED | 92 ( 4.8) |
| Less than high school | 1750 (92.3) |
| More than high school | 55 ( 2.9) |
| bmxbmi_cat_perc (%) |  |
| Underweight | 40 ( 2.1) |
| Healthy Weight | 1149 (60.5) |
| Overweight | 351 (18.5) |
| Obese | 359 (18.9) |

Background participant characteristics: Descriptives of the continuous variables.

|  | Overall |
| --- | --- |
| n | 1899 |
| ridageyr (mean (SD)) | 14.97 (2.00) |
| indfmpir (mean (SD)) | 2.07 (1.54) |

Background participant characteristics: Descriptives of the physical activity variable.

|  | Overall |
| --- | --- |
| n | 1899 |
| physical_act = Yes (%) | 1294 (70.9) |

## Weighted analysis: Demographics — 2 yr weights

Study participants by sex: Weighted statistics (percentage [95% CI]).

| var_name | values | perc_ci_95 |
| --- | --- | --- |
| sex | Female | 49.3 [46.8%, 51.7%] |
| sex | Male | 50.7 [48.3%, 53.2%] |

Demographic characteristics of study participants overall and by sex: Weighted statistics (mean (se) and median [iqr] presented for the continuous variables and percentage [95% CI] for the categorical variables).

| summary | values | Overall | Female | Male | p_val |
| --- | --- | --- | --- | --- | --- |
| Age at Screening Adjudicated |  | 15 (0.1) \| 15 [13, 17] | 15 (0.1) \| 15 [13, 17] | 14.9 (0.1) \| 15 [13, 17] | 0.275 |
| Family PIR |  | 2.6 (0.1) \| 2.5 [1.1, 4.1] | 2.6 (0.1) \| 2.5 [1.1, 4.1] | 2.6 (0.1) \| 2.4 [1.2, 4.2] | 0.834 |
| Ethnicity | Mexican American | 11 [5.4%, 16.5%] | 10.6 [5.1%, 16%] | 11.3 [5.6%, 17.1%] | 0.564 |
| Ethnicity | Non-Hispanic Black | 14.7 [9.7%, 19.8%] | 14.3 [9.5%, 19%] | 15.2 [9.6%, 20.7%] |  |
| Ethnicity | Non-Hispanic White | 64.5 [54.8%, 74.2%] | 66.1 [56.1%, 76%] | 63 [53%, 73.1%] |  |
| Ethnicity | Other | 4.9 [2.9%, 6.8%] | 4.8 [2.2%, 7.4%] | 5 [2.4%, 7.5%] |  |
| Ethnicity | Other Hispanic | 4.9 [2.3%, 7.4%] | 4.2 [1.3%, 7.2%] | 5.5 [3%, 8%] |  |
| Educational level | High school diploma including GED | 5.9 [2.8%, 9%] | 5.1 [2.7%, 7.6%] | 6.7 [2.2%, 11.1%] | 0.474 |
| Educational level | Less than high school | 90.5 [86.9%, 94.1%] | 90.8 [87.6%, 94%] | 90.3 [85.1%, 95.4%] |  |
| Educational level | More than high school | 3.6 [1.9%, 5.2%] | 4.1 [2%, 6.2%] | 3.1 [1%, 5.2%] |  |
| Smokers at home | No | 74.9 [69.6%, 80.1%] | 74 [66.3%, 81.7%] | 75.7 [70.7%, 80.7%] | 0.628 |
| Smokers at home | Yes | 25.1 [19.9%, 30.4%] | 26 [18.3%, 33.7%] | 24.3 [19.3%, 29.3%] |  |

## Weighted analysis: Nutrition day 1 Variables — nutrition weights

Nutrition dietary variables of study participants overall and by sex: Weighted statistics (mean (SD) and median[IQR]).

| summary | Overall | Female | Male | p_val |
| --- | --- | --- | --- | --- |
| Energy (kcal) | 2334.8 (47) \| 2213 [1615, 2826] | 1994.1 (37.5) \| 1918 [1441, 2418] | 2648.2 (76.7) \| 2494 [1820, 3275] | 0.000 |
| Protein (gm) | 81 (2) \| 75.8 [51.9, 99.2] | 67.4 (1.4) \| 63.1 [44.3, 83.2] | 93.6 (3.4) \| 86.6 [63, 120.3] | 0.000 |
| Carbohydrate (gm) | 310 (6.2) \| 287.7 [212.3, 377.7] | 265.8 (5.5) \| 251.9 [190.9, 325.6] | 350.7 (9.8) \| 326.6 [243.8, 432.1] | 0.000 |
| Total sugars (gm) | 157.6 (2.9) \| 146.6 [100.8, 200.6] | 136.2 (3.6) \| 127.8 [87.5, 170.9] | 177.3 (4.9) \| 159.5 [114, 227.6] | 0.000 |
| Dietary fiber (gm) | 13.6 (0.4) \| 12.1 [7.8, 17.4] | 12 (0.4) \| 10.3 [7.1, 14.7] | 15.1 (0.5) \| 13.7 [8.5, 19.3] | 0.000 |
| Total fat (gm) | 87.3 (2) \| 78.1 [54.9, 110.9] | 75.7 (2) \| 69 [49.1, 96.4] | 98 (3.2) \| 90.2 [62, 122.7] | 0.000 |
| Total saturated fatty acids (gm) | 29.9 (0.7) \| 27.4 [17.9, 38] | 25.7 (0.7) \| 23.6 [15.3, 33.7] | 33.9 (1.2) \| 30.5 [20.9, 44.4] | 0.000 |
| Total monounsaturated fatty acids (gm) | 32.5 (0.8) \| 29.2 [20, 42.4] | 27.9 (0.8) \| 25.7 [17.6, 34.9] | 36.8 (1.2) \| 33.8 [22.6, 47.8] | 0.000 |
| Total polyunsaturated fatty acids (gm) | 17.9 (0.5) \| 14.7 [9.3, 23] | 16.2 (0.5) \| 13.2 [8.6, 20.2] | 19.5 (0.8) \| 16.7 [10.1, 25.1] | 0.000 |
| Cholesterol (mg) | 245.7 (6.3) \| 196 [122, 312] | 202.5 (8.1) \| 167 [106, 245] | 285.5 (9.5) \| 225 [143, 352] | 0.000 |
| Vitamin E as alpha-tocopherol (mg) | 6.9 (0.3) \| 5.1 [3.4, 8.3] | 6.1 (0.2) \| 4.7 [3.1, 7.8] | 7.6 (0.5) \| 5.7 [3.6, 8.9] | 0.006 |
| Added alpha-tocopherol (Vitamin E) (mg) | 0.2 (0) \| 0 [0, 0] | 0.3 (0.1) \| 0 [0, 0] | 0.2 (0.1) \| 0 [0, 0] | 0.190 |
| Retinol (mcg) | 458.2 (21.6) \| 379 [188, 609] | 396 (22.4) \| 336 [160, 539] | 515.4 (24.7) \| 428 [215, 676] | 0.000 |
| Vitamin A, RAE (mcg) | 557 (24.2) \| 452 [244, 758] | 496 (24.4) \| 420 [217, 671] | 613.1 (28.6) \| 494 [287, 826] | 0.000 |
| Alpha-carotene (mcg) | 174.5 (18.2) \| 20 [4, 59] | 181.7 (22.7) \| 20 [5, 65] | 167.9 (25.5) \| 19 [2, 58] | 0.269 |
| Beta-carotene (mcg) | 1025.3 (92.6) \| 385 [176, 869] | 1050.5 (136.1) \| 350 [153, 819] | 1002.1 (104.7) \| 420 [204, 889] | 0.167 |
| Beta-cryptoxanthin (mcg) | 151.2 (18.8) \| 16 [2, 121] | 121.7 (10.6) \| 15 [2, 116] | 178.3 (31.6) \| 16 [2, 124] | 0.472 |
| Lycopene (mcg) | 6499.5 (393) \| 1840 [4, 9088] | 4600 (322.5) \| 932 [0, 6546] | 8246.6 (580.2) \| 3080 [311, 11055] | 0.000 |
| Lutein + zeaxanthin (mcg) | 858 (69.6) \| 444 [200, 930] | 857 (107.2) \| 422 [194, 904] | 858.9 (72.7) \| 454 [218, 997] | 0.406 |
| Thiamin (Vitamin B1) (mg) | 1.8 (0.1) \| 1.6 [1.1, 2.3] | 1.5 (0.1) \| 1.4 [1, 1.8] | 2.1 (0.1) \| 1.9 [1.2, 2.6] | 0.000 |
| Riboflavin (Vitamin B2) (mg) | 2.3 (0.1) \| 2 [1.4, 2.9] | 1.9 (0.1) \| 1.8 [1.1, 2.6] | 2.6 (0.1) \| 2.3 [1.6, 3.4] | 0.000 |
| Niacin (mg) | 23.4 (0.7) \| 21.4 [14.2, 29.1] | 19.8 (0.6) \| 17.9 [12.3, 25.3] | 26.7 (0.9) \| 24.5 [16.7, 33.4] | 0.000 |
| Vitamin B6 (mg) | 1.8 (0.1) \| 1.6 [1, 2.4] | 1.5 (0.1) \| 1.3 [0.8, 1.9] | 2.1 (0.1) \| 1.9 [1.2, 2.8] | 0.000 |
| Total Folate (mcg) | 421.3 (13.3) \| 351 [231, 537] | 366.3 (12.4) \| 314 [212, 439] | 471.9 (15.8) \| 417 [268, 600] | 0.000 |
| Folic acid (mcg) | 247.2 (9.9) \| 187 [110, 312] | 216.6 (11.1) \| 166 [89, 273] | 275.3 (11) \| 211 [131, 352] | 0.000 |
| Food folate (mcg) | 174.1 (4.1) \| 145 [99, 219] | 149.6 (3.4) \| 125 [92, 190] | 196.7 (7.6) \| 166 [111, 245] | 0.000 |
| Folate, DFE (mcg) | 594.5 (20.1) \| 480 [312, 750] | 518.1 (20) \| 426 [286, 638] | 664.8 (23) \| 582 [371, 843] | 0.000 |
| Vitamin B12 (mcg) | 5.4 (0.2) \| 4.6 [2.5, 7.1] | 4.4 (0.2) \| 3.4 [2.1, 6] | 6.3 (0.3) \| 5.5 [3.3, 8.2] | 0.000 |
| Added vitamin B12 (mcg) | 1 (0.1) \| 0 [0, 1.6] | 0.9 (0.1) \| 0 [0, 1.2] | 1.2 (0.1) \| 0 [0, 1.7] | 0.154 |
| Vitamin C (mg) | 89.1 (6.1) \| 47.3 [17.2, 119.6] | 75.4 (4.4) \| 43.8 [15.3, 106.7] | 101.7 (9.7) \| 52.6 [19.1, 133.3] | 0.019 |
| Vitamin K (mcg) | 62.5 (4.2) \| 40 [23.2, 69.1] | 60.3 (5.9) \| 36.4 [21.1, 63.9] | 64.5 (4) \| 45.7 [27.3, 72.2] | 0.001 |
| Calcium (mg) | 1044.6 (38.2) \| 881 [584, 1410] | 890.5 (37.6) \| 791 [504, 1148] | 1186.3 (48.2) \| 1019 [628, 1589] | 0.000 |
| Phosphorus (mg) | 1367.4 (36.1) \| 1262 [866, 1735] | 1158.7 (31.1) \| 1072 [774, 1425] | 1559.3 (53.2) \| 1413 [1041, 1932] | 0.000 |
| Magnesium (mg) | 247.9 (6.9) \| 218 [156, 316] | 212.2 (6.2) \| 189 [144, 258] | 280.7 (10.4) \| 261 [180, 340] | 0.000 |
| Iron (mg) | 16.7 (0.5) \| 14.3 [10.1, 21.1] | 14.1 (0.5) \| 12.4 [8.7, 16.7] | 19.1 (0.6) \| 16.6 [11.3, 24.3] | 0.000 |
| Zinc (mg) | 12.5 (0.4) \| 11 [7.1, 15.8] | 10.3 (0.4) \| 9.3 [6.1, 13.3] | 14.5 (0.5) \| 13 [8.5, 18.6] | 0.000 |
| Copper (mg) | 1.2 (0) \| 1 [0.7, 1.4] | 1 (0) \| 0.9 [0.6, 1.2] | 1.3 (0.1) \| 1.2 [0.8, 1.7] | 0.000 |
| Sodium (mg) | 3558.5 (106.4) \| 3172 [2238, 4524] | 3000.7 (77.3) \| 2740 [2007, 3637] | 4071.6 (170.9) \| 3775 [2558, 5170] | 0.000 |
| Potassium (mg) | 2432.3 (77.9) \| 2220 [1503, 3078] | 2069.5 (39.5) \| 1913 [1369, 2614] | 2766 (120.4) \| 2564 [1767, 3663] | 0.000 |
| Selenium (mcg) | 105.8 (2.8) \| 96 [65.9, 132.8] | 88.8 (2.5) \| 82.4 [57.7, 110.3] | 121.4 (4.3) \| 112.8 [78, 151.9] | 0.000 |
| Caffeine (mg) | 58.2 (3) \| 30 [1, 84] | 55.1 (5) \| 29 [1, 75] | 61.1 (3.9) \| 31 [1, 93] | 0.449 |
| Theobromine (mg) | 50.3 (4.3) \| 5 [0, 58] | 43.5 (3.1) \| 5 [0, 58] | 56.5 (7.4) \| 2 [0, 58] | 0.802 |
| Alcohol (gm) | 1.3 (0.2) \| 0 [0, 0] | 0.5 (0.2) \| 0 [0, 0] | 2 (0.4) \| 0 [0, 0] | 0.889 |
| Moisture (gm) | 1614.4 (31.2) \| 1455.9 [1070.2, 1969.6] | 1355.2 (30.2) \| 1259.8 [974.7, 1678.3] | 1852.9 (51.1) \| 1699.1 [1244.1, 2321.8] | 0.000 |
| SFA 4:0 (Butanoic) (gm) | 0.7 (0) \| 0.6 [0.3, 1] | 0.6 (0) \| 0.5 [0.2, 0.9] | 0.8 (0) \| 0.7 [0.3, 1.1] | 0.004 |
| SFA 6:0 (Hexanoic) (gm) | 0.4 (0) \| 0.3 [0.1, 0.5] | 0.3 (0) \| 0.3 [0.1, 0.5] | 0.4 (0) \| 0.4 [0.2, 0.6] | 0.004 |
| SFA 8:0 (Octanoic) (gm) | 0.3 (0) \| 0.2 [0.1, 0.4] | 0.2 (0) \| 0.2 [0.1, 0.3] | 0.3 (0) \| 0.3 [0.1, 0.4] | 0.002 |
| SFA 10:0 (Decanoic) (gm) | 0.5 (0) \| 0.4 [0.2, 0.7] | 0.5 (0) \| 0.4 [0.2, 0.6] | 0.6 (0) \| 0.5 [0.3, 0.8] | 0.003 |
| SFA 12:0 (Dodecanoic) (gm) | 0.7 (0) \| 0.5 [0.3, 0.9] | 0.7 (0) \| 0.4 [0.2, 0.8] | 0.8 (0) \| 0.6 [0.3, 1] | 0.000 |
| SFA 14:0 (Tetradecanoic) (gm) | 2.7 (0.1) \| 2.3 [1.3, 3.6] | 2.3 (0.1) \| 1.9 [1.1, 3.1] | 3 (0.1) \| 2.6 [1.5, 4.1] | 0.000 |
| SFA 16:0 (Hexadecanoic) (gm) | 16.1 (0.4) \| 14.7 [9.9, 20.5] | 13.9 (0.4) \| 12.8 [8.7, 18.1] | 18.2 (0.6) \| 16.5 [11.3, 24.1] | 0.000 |
| SFA 18:0 (Octadecanoic) (gm) | 7.6 (0.2) \| 6.9 [4.6, 9.9] | 6.5 (0.2) \| 5.8 [3.9, 8.5] | 8.7 (0.3) \| 8.1 [5.4, 11.5] | 0.000 |
| MFA 16:1 (Hexadecenoic) (gm) | 1.4 (0) \| 1.2 [0.7, 1.9] | 1.1 (0) \| 1 [0.6, 1.5] | 1.6 (0.1) \| 1.4 [0.9, 2.1] | 0.000 |
| MFA 18:1 (Octadecenoic) (gm) | 30.4 (0.8) \| 27.2 [18.5, 39.7] | 26.1 (0.8) \| 24.1 [16.3, 32.3] | 34.3 (1.1) \| 31.3 [21.1, 44.3] | 0.000 |
| MFA 20:1 (Eicosenoic) (gm) | 0.2 (0) \| 0.2 [0.1, 0.3] | 0.2 (0) \| 0.2 [0.1, 0.2] | 0.2 (0) \| 0.2 [0.1, 0.3] | 0.000 |
| MFA 22:1 (Docosenoic) (gm) | 0 (0) \| 0 [0, 0] | 0 (0) \| 0 [0, 0] | 0 (0) \| 0 [0, 0] | 0.046 |
| PFA 18:2 (Octadecadienoic) (gm) | 15.9 (0.5) \| 12.9 [8.3, 20.4] | 14.3 (0.5) \| 11.5 [7.5, 17.7] | 17.4 (0.8) \| 14.5 [9, 22.4] | 0.001 |
| PFA 18:3 (Octadecatrienoic) (gm) | 1.5 (0) \| 1.3 [0.7, 1.9] | 1.4 (0.1) \| 1.1 [0.7, 1.7] | 1.6 (0.1) \| 1.4 [0.8, 2.1] | 0.000 |
| PFA 18:4 (Octadecatetraenoic) (gm) | 0 (0) \| 0 [0, 0] | 0 (0) \| 0 [0, 0] | 0 (0) \| 0 [0, 0] | 0.983 |
| PFA 20:4 (Eicosatetraenoic) (gm) | 0.1 (0) \| 0.1 [0, 0.1] | 0.1 (0) \| 0.1 [0, 0.1] | 0.1 (0) \| 0.1 [0, 0.2] | 0.001 |
| PFA 20:5 (Eicosapentaenoic) (gm) | 0 (0) \| 0 [0, 0] | 0 (0) \| 0 [0, 0] | 0 (0) \| 0 [0, 0] | 0.104 |
| PFA 22:5 (Docosapentaenoic) (gm) | 0 (0) \| 0 [0, 0] | 0 (0) \| 0 [0, 0] | 0 (0) \| 0 [0, 0] | 0.089 |
| PFA 22:6 (Docosahexaenoic) (gm) | 0 (0) \| 0 [0, 0] | 0 (0) \| 0 [0, 0] | 0 (0) \| 0 [0, 0] | 0.054 |

## Weighted analysis: Laboratory Categorical and Continuous Variables — mec weights

Laboratory characteristics of study participants overall and by sex: Weighted statistics (mean (SD) and median [IQR])).

|  |  |  |  |  |
| --- | --- | --- | --- | --- |
| summary | Overall | Female | Male | p_val |
| Basophils number | 0 (0) \| 0 [0, 0.1] | 0 (0) \| 0 [0, 0.1] | 0 (0) \| 0 [0, 0.1] | 0.060 |
| Eosinophils number | 0.2 (0) \| 0.1 [0.1, 0.2] | 0.2 (0) \| 0.1 [0.1, 0.2] | 0.2 (0) \| 0.2 [0.1, 0.3] | 0.006 |
| Lymphocyte number | 2.2 (0) \| 2.1 [1.7, 2.6] | 2.2 (0) \| 2.2 [1.8, 2.6] | 2.2 (0) \| 2.1 [1.7, 2.5] | 0.148 |
| Monocyte number | 0.6 (0) \| 0.5 [0.4, 0.7] | 0.5 (0) \| 0.5 [0.4, 0.6] | 0.6 (0) \| 0.5 [0.4, 0.7] | 0.125 |
| Segmented neutrophils number | 4.1 (0.1) \| 3.8 [2.9, 4.9] | 4.4 (0.1) \| 4.2 [3.1, 5.3] | 3.8 (0.1) \| 3.4 [2.7, 4.6] | 0.000 |
| Basophils percent (%) | 0.7 (0) \| 0.6 [0.4, 0.8] | 0.6 (0) \| 0.6 [0.4, 0.8] | 0.7 (0) \| 0.6 [0.4, 0.8] | 0.165 |
| Eosinophils percent (%) | 2.9 (0.1) \| 2.2 [1.3, 3.7] | 2.5 (0.1) \| 1.8 [1.2, 3.2] | 3.3 (0.2) \| 2.5 [1.7, 4.1] | 0.000 |
| Hematocrit (%) | 42.3 (0.2) \| 42.2 [39.8, 44.9] | 40.1 (0.2) \| 40.2 [38.3, 42.1] | 44.5 (0.2) \| 44.5 [42.2, 46.6] | 0.000 |
| Hemoglobin (g/dL) | 14.4 (0.1) \| 14.4 [13.6, 15.2] | 13.6 (0.1) \| 13.7 [13.1, 14.3] | 15.1 (0.1) \| 15 [14.4, 15.8] | 0.000 |
| Lymphocyte percent (%) | 32.2 (0.2) \| 32.3 [26.5, 37.7] | 31.4 (0.3) \| 31.2 [25.6, 36.5] | 33 (0.3) \| 32.8 [27.1, 38.7] | 0.001 |
| Mean cell hemoglobin (pg) | 29.7 (0.1) \| 29.8 [28.8, 30.8] | 29.7 (0.1) \| 29.9 [29, 31] | 29.6 (0.1) \| 29.8 [28.7, 30.8] | 0.138 |
| Mean cell volume (fL) | 87.5 (0.2) \| 87.9 [85.1, 90.2] | 87.5 (0.2) \| 87.9 [85.4, 90.6] | 87.5 (0.2) \| 87.9 [84.6, 90.1] | 0.241 |
| Monocyte percent (%) | 8.2 (0.1) \| 8 [6.6, 9.4] | 7.7 (0.1) \| 7.5 [6.2, 8.9] | 8.6 (0.1) \| 8.4 [7.2, 9.7] | 0.000 |
| Mean platelet volume (fL) | 8 (0) \| 8 [7.5, 8.5] | 8 (0) \| 8 [7.5, 8.4] | 8.1 (0) \| 8 [7.6, 8.6] | 0.152 |
| Segmented neutrophils percent (%) | 56 (0.2) \| 56.2 [49.4, 62.8] | 57.8 (0.3) \| 58.3 [52.1, 64.3] | 54.4 (0.3) \| 54.3 [47.4, 61.1] | 0.000 |
| Platelet count SI (1000 cells/uL) | 282.2 (2.4) \| 275 [237, 319] | 295.1 (3.1) \| 291 [247, 331] | 269.9 (3) \| 264 [227, 304] | 0.000 |
| Red blood cell count (million cells/uL) | 4.8 (0) \| 4.8 [4.5, 5.1] | 4.6 (0) \| 4.6 [4.4, 4.8] | 5.1 (0) \| 5.1 [4.9, 5.3] | 0.000 |
| Red cell distribution width (%) | 12.4 (0) \| 12.3 [12, 12.7] | 12.4 (0) \| 12.2 [11.9, 12.6] | 12.4 (0) \| 12.3 [12, 12.7] | 0.061 |
| White blood cell count (1000 cells/uL) | 7.1 (0.1) \| 6.9 [5.6, 8.2] | 7.4 (0.1) \| 7.2 [5.8, 8.6] | 6.8 (0.1) \| 6.6 [5.4, 7.8] | 0.001 |
| Albumin (g/L) | 44.3 (0.1) \| 44 [42, 46] | 43.3 (0.2) \| 43 [41, 45] | 45.3 (0.2) \| 45 [43, 47] | 0.000 |
| Blood urea nitrogen (mmol/L) | 3.5 (0) \| 3.2 [2.9, 3.9] | 3.2 (0.1) \| 3.2 [2.5, 3.9] | 3.7 (0.1) \| 3.6 [2.9, 4.3] | 0.000 |
| Total calcium (mmol/L) | 2.4 (0) \| 2.5 [2.4, 2.5] | 2.4 (0) \| 2.4 [2.4, 2.5] | 2.5 (0) \| 2.5 [2.4, 2.5] | 0.000 |
| Cholesterol (mmol/L) | 4.2 (0) \| 4.1 [3.7, 4.7] | 4.3 (0) \| 4.2 [3.8, 4.7] | 4.1 (0) \| 4.1 [3.6, 4.6] | 0.001 |
| Globulin (g/L) | 28.6 (0.2) \| 28 [26, 31] | 29.3 (0.2) \| 29 [27, 32] | 27.9 (0.2) \| 28 [26, 30] | 0.000 |
| Glucose, serum (mmol/L) | 4.8 (0) \| 4.8 [4.5, 5] | 4.7 (0) \| 4.7 [4.4, 4.9] | 4.9 (0) \| 4.9 [4.6, 5.1] | 0.000 |
| Iron, refigerated (umol/L) | 15.6 (0.3) \| 15 [10.6, 20.2] | 14.7 (0.5) \| 13.8 [9, 19.3] | 16.5 (0.3) \| 15.9 [11.5, 20.8] | 0.008 |
| Phosphorus (mmol/L) | 1.4 (0) \| 1.4 [1.3, 1.6] | 1.4 (0) \| 1.4 [1.3, 1.5] | 1.5 (0) \| 1.5 [1.3, 1.6] | 0.000 |
| Bilirubin, total (umol/L) | 13.3 (0.3) \| 12 [10.3, 15.4] | 12 (0.4) \| 10.3 [8.6, 13.7] | 14.6 (0.4) \| 13.7 [10.3, 17.1] | 0.000 |
| Total protein (g/L) | 72.9 (0.2) \| 73 [70, 75] | 72.6 (0.3) \| 72 [69, 75] | 73.1 (0.3) \| 73 [70, 76] | 0.086 |
| Triglycerides (mmol/L) | 0.9 (0) \| 0.8 [0.6, 1.1] | 0.9 (0) \| 0.7 [0.5, 1] | 1 (0) \| 0.8 [0.6, 1.1] | 0.013 |
| Uric acid (umol/L) | 305.3 (2.4) \| 297.4 [249.8, 350.9] | 267.2 (3.5) \| 261.7 [232, 303.3] | 341.5 (2.2) \| 339 [297.4, 380.7] | 0.000 |
| Alkaline phosphotase (U/L) | 151.1 (4.8) \| 110 [73, 214] | 104.7 (3.9) \| 81 [63, 123] | 195.1 (6.5) \| 171 [101, 267] | 0.000 |
| Aspartate aminotransferase AST (U/L) | 24.3 (0.4) \| 22 [20, 26] | 21.9 (0.6) \| 20 [18, 23] | 26.5 (0.6) \| 24 [21, 28] | 0.000 |
| Alanine aminotransferase ALT (U/L) | 19.2 (0.3) \| 17 [14, 21] | 16.9 (0.4) \| 15 [13, 18] | 21.4 (0.6) \| 18 [15, 23] | 0.000 |
| Bicarbonate (mmol/L) | 24.3 (0.1) \| 24 [23, 26] | 23.9 (0.1) \| 24 [23, 25] | 24.7 (0.1) \| 25 [23, 26] | 0.000 |
| Chloride (mmol/L) | 104 (0.1) \| 104 [103, 105] | 104.4 (0.1) \| 104 [103, 106] | 103.5 (0.1) \| 103 [102, 105] | 0.000 |
| Creatinine (mg/dL) | 0.8 (0) \| 0.7 [0.6, 0.8] | 0.7 (0) \| 0.7 [0.6, 0.8] | 0.8 (0) \| 0.8 [0.7, 0.9] | 0.000 |
| Gamma glutamyl transferase (U/L) | 13.9 (0.2) \| 12 [10, 16] | 12 (0.3) \| 11 [9, 14] | 15.6 (0.3) \| 14 [11, 18] | 0.000 |
| Potassium (mmol/L) | 4 (0) \| 4 [3.8, 4.1] | 3.9 (0) \| 3.9 [3.8, 4.1] | 4 (0) \| 4 [3.9, 4.2] | 0.000 |
| Lactate dehydrogenase LDH (U/L) | 133.9 (2.3) \| 128 [112, 149] | 128.2 (3.5) \| 122 [109, 139] | 139.3 (1.6) \| 137 [117, 156] | 0.000 |
| Sodium (mmol/L) | 139.1 (0.1) \| 139 [138, 140] | 138.9 (0.1) \| 139 [138, 140] | 139.3 (0.1) \| 139 [138, 140] | 0.000 |
| Osmolality (mmol/Kg) | 276.1 (0.2) \| 276 [274, 278] | 275.3 (0.2) \| 275 [273, 277] | 276.7 (0.2) \| 277 [275, 278] | 0.000 |
| Albumin, urine (mg/L) SI | 29.7 (2.4) \| 8.9 [4.9, 18.9] | 31.9 (2.8) \| 10.3 [5.7, 23.4] | 27.5 (4) \| 8 [4.6, 16] | 0.000 |
| Cotinine (ng/mL) | 19.4 (2.8) \| 0.1 [0, 1.1] | 16.8 (2.8) \| 0.1 [0, 1] | 21.8 (4) \| 0.1 [0, 1.3] | 0.345 |
| Glycohemoglobin (%) | 5.2 (0) \| 5.2 [5, 5.3] | 5.1 (0) \| 5.1 [5, 5.3] | 5.2 (0) \| 5.2 [5, 5.3] | 0.005 |
| BMI SDS | 0.6 (0.1) \| 0.6 [-0.1, 1.4] | 0.6 (0.1) \| 0.6 [0, 1.4] | 0.6 (0.1) \| 0.6 [-0.2, 1.5] | 0.922 |
| BMI SDS percentile | 0.7 (0) \| 0.7 [0.5, 0.9] | 0.7 (0) \| 0.7 [0.5, 0.9] | 0.7 (0) \| 0.7 [0.4, 0.9] | 0.946 |

Physical activity and BMI (cat.): Weighted statistics (percentage [95% CI])).

| summary | values | Overall | Female | Male | p_val |
| --- | --- | --- | --- | --- | --- |
| Physical activity | No | 25.3 [22.7%, 27.9%] | 33.5 [30.5%, 36.5%] | 17.6 [13.5%, 21.7%] | 0.000 |
| Physical activity | Yes | 74.7 [72.1%, 77.3%] | 66.5 [63.5%, 69.5%] | 82.4 [78.3%, 86.5%] |  |
| BMI categories | Underweight | 2.2 [1.1%, 3.2%] | 1.4 [0.2%, 2.6%] | 2.9 [1.7%, 4.2%] | 0.085 |
| BMI categories | Healthy Weight | 61.4 [55.7%, 67.1%] | 64.9 [57.2%, 72.5%] | 58.1 [52.2%, 63.9%] |  |
| BMI categories | Overweight | 18.4 [15.3%, 21.5%] | 16.8 [12.2%, 21.4%] | 19.9 [16.7%, 23.1%] |  |
| BMI categories | Obese | 18.1 [14.1%, 22.1%] | 16.9 [11.8%, 22.1%] | 19.1 [14.9%, 23.4%] |  |

## QQ-plots


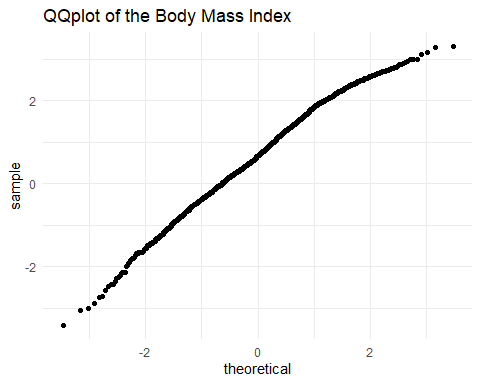


# 2013-2014

## Descriptives - Background: unweighted statistics 2013-2014

Background participant characteristics: Descriptives of the categorical variables.

|  | Overall |
| --- | --- |
| n | 1224 |
| sex = Male (%) | 624 (51.0) |
| smoker_home = Yes (%) | 277 (22.8) |
| ethnicity (%) |  |
| Mexican American | 287 (23.4) |
| Non-Hispanic Black | 301 (24.6) |
| Non-Hispanic White | 306 (25.0) |
| Other | 203 (16.6) |
| Other Hispanic | 127 (10.4) |
| edu (%) |  |
| High school diploma including GED | 78 ( 6.4) |
| Less than high school | 1120 (91.6) |
| More than high school | 25 ( 2.0) |
| bmxbmi_cat_perc (%) |  |
| Underweight | 32 ( 2.6) |
| Healthy Weight | 702 (57.4) |
| Overweight | 232 (19.0) |
| Obese | 258 (21.1) |

Background participant characteristics: Descriptives of the continuous variables.

|  | Overall |
| --- | --- |
| n | 1224 |
| ridageyr (mean (SD)) | 14.97 (2.00) |
| indfmpir (mean (SD)) | 2.00 (1.55) |

Background participant characteristics: Descriptives of the physical activity variable

|  | Overall |
| --- | --- |
| n | 1224 |
| physical_act = Yes (%) | 723 (60.5) |

## Weighted analysis: Demographics — 2 yr weights

Study participants by sex: Weighted statistics (percentage [95% CI]).

| var_name | values | perc_ci_95 |
| --- | --- | --- |
| sex | Female | 48.2 [44.3%, 52.1%] |
| sex | Male | 51.8 [47.9%, 55.7%] |

Demographic characteristics of study participants overall and by sex: Weighted statistics (mean (SE) and median [IQR] presented for the continuous variables and percentage [95% CI] for the categorical variables).

| summary | values | Overall | Female | Male | p_val |
| --- | --- | --- | --- | --- | --- |
| Age in years at screening |  | 15 (0.1) \| 15 [13, 17] | 15.1 (0.1) \| 15 [14, 17] | 15 (0.1) \| 15 [13, 17] | 0.641 |
| Ratio of family income to poverty |  | 2.4 (0.1) \| 2.2 [1, 3.8] | 2.4 (0.1) \| 2.1 [1, 3.6] | 2.5 (0.2) \| 2.2 [1, 4] | 0.633 |
| Ethnicity | Mexican American | 15.4 [8.9%, 22%] | 15.2 [9.3%, 21%] | 15.6 [8.1%, 23.2%] | 0.936 |
| Ethnicity | Non-Hispanic Black | 14.3 [9.6%, 19%] | 14 [9.3%, 18.8%] | 14.5 [9%, 20%] |  |
| Ethnicity | Non-Hispanic White | 54.2 [43.3%, 65%] | 54.3 [42.5%, 66.1%] | 54 [43.1%, 64.8%] |  |
| Ethnicity | Other | 9.2 [6.6%, 11.8%] | 9.8 [6.8%, 12.8%] | 8.7 [5.1%, 12.3%] |  |
| Ethnicity | Other Hispanic | 6.9 [4.4%, 9.4%] | 6.7 [3.7%, 9.6%] | 7.1 [4.2%, 10%] |  |
| Educational level | High school diploma including GED | 6.6 [4.4%, 8.8%] | 6.1 [2.1%, 10%] | 7.2 [4.5%, 9.8%] | 0.751 |
| Educational level | Less than high school | 91.8 [89.3%, 94.3%] | 92.4 [87.9%, 96.9%] | 91.2 [88.6%, 93.8%] |  |
| Educational level | More than high school | 1.6 [0.7%, 2.5%] | 1.5 [0.4%, 2.7%] | 1.6 [0.7%, 2.6%] |  |
| Smokers at home | No | 76.4 [70.4%, 82.5%] | 77.6 [70.5%, 84.7%] | 75.3 [68.7%, 81.9%] | 0.462 |
| Smokers at home | Yes | 23.6 [17.5%, 29.6%] | 22.4 [15.3%, 29.5%] | 24.7 [18.1%, 31.3%] |  |

## Weighted analysis: Nutrition day 1 Variables — nutrition weights

Nutrition dietary variables of study participants overall and by sex: Weighted statistics (mean (SD) and median [IQR]).

| summary | Overall | Female | Male | p_val |
| --- | --- | --- | --- | --- |
| Energy (kcal) | 2023.9 (36) \| 1833 [1356, 2474] | 1661.7 (51.9) \| 1509 [1169, 2082] | 2371.2 (43.5) \| 2137 [1665, 2871] | 0.000 |
| Protein (gm) | 77.9 (2.1) \| 67.9 [49, 96.7] | 60.2 (2.3) \| 58.2 [39.8, 75.8] | 94.9 (3.8) \| 81.6 [59.9, 113.1] | 0.000 |
| Carbohydrate (gm) | 259.1 (5.2) \| 238.2 [170.1, 323.8] | 217.4 (5.6) \| 200.6 [149.6, 271.4] | 299.1 (8.4) \| 269.8 [208.7, 359.2] | 0.000 |
| Total sugars (gm) | 120.6 (2.9) \| 102.2 [70.4, 150.7] | 99.2 (2.9) \| 88.9 [62.4, 125.8] | 141.1 (4.9) \| 124.5 [85.8, 174.5] | 0.000 |
| Dietary fiber (gm) | 14.5 (0.5) \| 12.6 [8.4, 18.5] | 12.4 (0.6) \| 10.8 [7.4, 16] | 16.5 (0.5) \| 14.2 [9.6, 19.9] | 0.000 |
| Total fat (gm) | 76.9 (1.8) \| 67.8 [46.6, 94.9] | 63.1 (2.8) \| 57.8 [36.8, 80.6] | 90 (1.7) \| 77.4 [57.9, 115.8] | 0.000 |
| Total saturated fatty acids (gm) | 25.8 (0.7) \| 22.5 [14.9, 33] | 20.9 (1) \| 18.3 [11.4, 27] | 30.4 (0.8) \| 27.2 [17.4, 38.8] | 0.000 |
| Total monounsaturated fatty acids (gm) | 26 (0.7) \| 22.1 [14.8, 32.8] | 21 (1.1) \| 18.7 [11.9, 26.8] | 30.8 (0.9) \| 25.5 [18.3, 38.9] | 0.000 |
| Total polyunsaturated fatty acids (gm) | 17.6 (0.7) \| 14.7 [9.5, 22.6] | 15.2 (0.8) \| 12.6 [7.9, 20.3] | 19.9 (0.8) \| 16.9 [11.7, 24.4] | 0.000 |
| Cholesterol (mg) | 246.6 (9.1) \| 176 [102, 317] | 181.1 (8.7) \| 139 [83, 235] | 309.5 (16.6) \| 244 [135, 385] | 0.000 |
| Vitamin E as alpha-tocopherol (mg) | 8 (0.6) \| 6 [3.9, 9.4] | 6.6 (0.3) \| 5.1 [3, 8.5] | 9.3 (1.1) \| 7 [4.5, 9.8] | 0.000 |
| Added alpha-tocopherol (Vitamin E) (mg) | 0.7 (0.3) \| 0 [0, 0] | 0.4 (0.1) \| 0 [0, 0] | 1.1 (0.6) \| 0 [0, 0] | 0.145 |
| Retinol (mcg) | 450.9 (14.2) \| 387 [191, 609] | 361.8 (20.6) \| 311 [140, 503] | 536.4 (22.2) \| 460 [248, 732] | 0.000 |
| Vitamin A, RAE (mcg) | 570.9 (21.6) \| 486 [267, 744] | 495.4 (39.7) \| 414 [217, 689] | 643.3 (20.1) \| 557 [332, 819] | 0.001 |
| Alpha-carotene (mcg) | 269.2 (58.9) \| 19 [2, 79] | 296.4 (101.8) \| 17 [2, 77] | 243.2 (41.2) \| 23 [2, 79] | 0.245 |
| Beta-carotene (mcg) | 1279.2 (170.1) \| 418 [200, 1021] | 1428.9 (321.6) \| 352 [179, 984] | 1135.6 (108.6) \| 473 [226, 1030] | 0.105 |
| Beta-cryptoxanthin (mcg) | 69.1 (6.4) \| 19 [4, 88] | 64.7 (10.2) \| 15 [2, 63] | 73.3 (7.9) \| 27 [7, 92] | 0.019 |
| Lycopene (mcg) | 4713.3 (250.5) \| 1890 [4, 5365] | 3979.4 (270.2) \| 1750 [5, 4392] | 5416.9 (363.1) \| 2097 [0, 6535] | 0.193 |
| Lutein + zeaxanthin (mcg) | 921 (83.1) \| 491 [237, 940] | 903.8 (167.1) \| 411 [206, 845] | 937.6 (75.3) \| 562 [284, 1030] | 0.076 |
| Thiamin (Vitamin B1) (mg) | 1.7 (0.1) \| 1.4 [1, 2.1] | 1.3 (0) \| 1.2 [0.8, 1.6] | 2 (0.1) \| 1.7 [1.2, 2.4] | 0.000 |
| Riboflavin (Vitamin B2) (mg) | 2.1 (0.1) \| 1.8 [1.2, 2.6] | 1.7 (0.1) \| 1.5 [1, 2.1] | 2.5 (0.1) \| 2.2 [1.4, 3.1] | 0.000 |
| Niacin (mg) | 25.3 (0.7) \| 21.3 [14, 30.8] | 19.5 (0.8) \| 18.1 [11.8, 23.8] | 30.9 (1.2) \| 26.4 [17.6, 38.1] | 0.000 |
| Vitamin B6 (mg) | 2 (0.1) \| 1.6 [1, 2.5] | 1.5 (0.1) \| 1.3 [0.9, 1.9] | 2.5 (0.1) \| 2 [1.2, 3.1] | 0.000 |
| Total folate (mcg) | 389.5 (13.9) \| 316 [201, 504] | 321 (13) \| 275 [176, 430] | 455.2 (22.2) \| 367 [242, 582] | 0.000 |
| Folic acid (mcg) | 217.8 (12.1) \| 151 [78, 270] | 175.5 (10) \| 127 [60, 232] | 258.3 (20.3) \| 183 [93, 299] | 0.001 |
| Food folate (mcg) | 171.7 (4.9) \| 145 [95, 219] | 145.5 (6.9) \| 127 [77, 187] | 196.8 (5.6) \| 162 [115, 250] | 0.000 |
| Folate, DFE (mcg) | 541.9 (22.1) \| 423 [262, 683] | 443.8 (19.3) \| 358 [229, 590] | 635.9 (36.1) \| 500 [323, 780] | 0.000 |
| Vitamin B12 (mcg) | 5.2 (0.2) \| 4 [2.3, 6.3] | 3.8 (0.1) \| 3.2 [1.9, 5.1] | 6.5 (0.4) \| 5 [2.8, 8.3] | 0.000 |
| Added vitamin B12 (mcg) | 1.5 (0.2) \| 0 [0, 1.7] | 1 (0.1) \| 0 [0, 1.4] | 2 (0.3) \| 0 [0, 2.3] | 0.200 |
| Vitamin C (mg) | 70.6 (3.4) \| 39.8 [14.2, 101.8] | 62.7 (4.2) \| 32.2 [12.5, 91.7] | 78.2 (5.3) \| 47.2 [16.7, 114.5] | 0.054 |
| Vitamin K (mcg) | 79.1 (6) \| 50.7 [29, 91.2] | 77.5 (11.4) \| 43.6 [22.7, 78.8] | 80.6 (4.7) \| 58.5 [33.3, 97.9] | 0.007 |
| Calcium (mg) | 1021.7 (26.3) \| 895 [561, 1323] | 832.5 (37.3) \| 777 [476, 1094] | 1203 (35.5) \| 1066 [680, 1546] | 0.000 |
| Phosphorus (mg) | 1352.4 (26.5) \| 1230 [898, 1663] | 1082 (39.2) \| 1051 [732, 1347] | 1611.6 (32.5) \| 1418 [1067, 1965] | 0.000 |
| Magnesium (mg) | 252.1 (5.4) \| 223 [158, 309] | 207.7 (6.9) \| 191 [137, 268] | 294.8 (7.2) \| 266 [187, 371] | 0.000 |
| Iron (mg) | 14.7 (0.4) \| 12.4 [8.2, 18.3] | 11.6 (0.4) \| 9.9 [6.8, 15] | 17.7 (0.8) \| 15 [10.1, 21.1] | 0.000 |
| Zinc (mg) | 11.1 (0.4) \| 9.1 [6.1, 13.6] | 8.2 (0.3) \| 7.3 [5.1, 10.6] | 13.8 (0.7) \| 11.3 [7.7, 16.2] | 0.000 |
| Copper (mg) | 1 (0) \| 0.9 [0.6, 1.2] | 0.8 (0) \| 0.7 [0.5, 1.1] | 1.1 (0) \| 1 [0.7, 1.4] | 0.000 |
| Sodium (mg) | 3367.2 (66.9) \| 2979 [2183, 4129] | 2788.2 (93.9) \| 2690 [1865, 3458] | 3922.2 (106.7) \| 3700 [2537, 5005] | 0.000 |
| Potassium (mg) | 2263.1 (47.3) \| 2031 [1441, 2889] | 1847.8 (66.4) \| 1742 [1191, 2380] | 2661.2 (48.4) \| 2453 [1722, 3281] | 0.000 |
| Selenium (mcg) | 108.1 (2.3) \| 97.3 [65, 135.8] | 85.8 (2.5) \| 78.3 [54.1, 110] | 129.6 (4.2) \| 117.4 [78.6, 157.7] | 0.000 |
| Caffeine (mg) | 46.7 (4.5) \| 8 [0, 57] | 44.1 (7.8) \| 8 [0, 47] | 49.1 (4.3) \| 8 [0, 67] | 0.317 |
| Theobromine (mg) | 40.8 (3.9) \| 2 [0, 51] | 33.9 (6) \| 0 [0, 37] | 47.5 (3.8) \| 8 [0, 65] | 0.011 |
| Alcohol (gm) | 0.9 (0.5) \| 0 [0, 0] | 0.6 (0.3) \| 0 [0, 0] | 1.3 (0.9) \| 0 [0, 0] | 0.601 |
| Moisture (gm) | 2181.5 (46.7) \| 1931.1 [1357.6, 2800.5] | 1963.1 (69.7) \| 1740.1 [1266.6, 2369.6] | 2390.8 (59.8) \| 2086.9 [1505.4, 3029.2] | 0.000 |
| SFA 4:0 (Butanoic) (gm) | 0.5 (0) \| 0.4 [0.2, 0.7] | 0.4 (0) \| 0.3 [0.1, 0.6] | 0.6 (0) \| 0.5 [0.2, 0.8] | 0.001 |
| SFA 6:0 (Hexanoic) (gm) | 0.3 (0) \| 0.2 [0.1, 0.4] | 0.3 (0) \| 0.2 [0.1, 0.4] | 0.4 (0) \| 0.3 [0.1, 0.5] | 0.002 |
| SFA 8:0 (Octanoic) (gm) | 0.3 (0) \| 0.2 [0.1, 0.3] | 0.2 (0) \| 0.2 [0.1, 0.3] | 0.3 (0) \| 0.2 [0.1, 0.4] | 0.004 |
| SFA 10:0 (Decanoic) (gm) | 0.5 (0) \| 0.4 [0.2, 0.7] | 0.4 (0) \| 0.3 [0.2, 0.6] | 0.6 (0) \| 0.5 [0.3, 0.8] | 0.002 |
| SFA 12:0 (Dodecanoic) (gm) | 0.8 (0) \| 0.5 [0.3, 1] | 0.7 (0) \| 0.5 [0.2, 0.8] | 0.9 (0.1) \| 0.6 [0.4, 1.1] | 0.008 |
| SFA 14:0 (Tetradecanoic) (gm) | 2.2 (0.1) \| 1.8 [0.9, 3] | 1.8 (0.1) \| 1.5 [0.7, 2.5] | 2.6 (0.1) \| 2 [1.3, 3.6] | 0.000 |
| SFA 16:0 (Hexadecanoic) (gm) | 14 (0.3) \| 12 [8.1, 17.3] | 11.3 (0.5) \| 10 [6.5, 15] | 16.6 (0.4) \| 14.2 [9.9, 21.2] | 0.000 |
| SFA 18:0 (Octadecanoic) (gm) | 6.1 (0.2) \| 5.4 [3.5, 7.7] | 4.9 (0.2) \| 4.4 [2.7, 6.3] | 7.3 (0.2) \| 6.3 [4.2, 9.7] | 0.000 |
| MFA 16:1 (Hexadecenoic) (gm) | 1 (0) \| 0.8 [0.5, 1.3] | 0.7 (0) \| 0.6 [0.4, 1] | 1.2 (0.1) \| 1 [0.6, 1.6] | 0.000 |
| MFA 18:1 (Octadecenoic) (gm) | 23.4 (0.7) \| 19.6 [13.5, 28.8] | 19.2 (1) \| 16.8 [11, 24.7] | 27.5 (0.9) \| 22.8 [16.3, 34.8] | 0.000 |
| MFA 20:1 (Eicosenoic) (gm) | 0.3 (0) \| 0.2 [0.1, 0.3] | 0.2 (0) \| 0.2 [0.1, 0.3] | 0.3 (0) \| 0.3 [0.2, 0.4] | 0.000 |
| MFA 22:1 (Docosenoic) (gm) | 0 (0) \| 0 [0, 0] | 0 (0) \| 0 [0, 0] | 0 (0) \| 0 [0, 0] | 0.158 |
| PFA 18:2 (Octadecadienoic) (gm) | 15.7 (0.6) \| 13.2 [8.3, 20.3] | 13.6 (0.7) \| 11.4 [6.9, 18.4] | 17.8 (0.7) \| 15.1 [10.4, 21.9] | 0.000 |
| PFA 18:3 (Octadecatrienoic) (gm) | 1.5 (0.1) \| 1.2 [0.7, 2] | 1.3 (0.1) \| 1.1 [0.6, 1.7] | 1.7 (0.1) \| 1.4 [0.9, 2.2] | 0.001 |
| PFA 18:4 (Octadecatetraenoic) (gm) | 0 (0) \| 0 [0, 0] | 0 (0) \| 0 [0, 0] | 0 (0) \| 0 [0, 0] | 0.189 |
| PFA 20:4 (Eicosatetraenoic) (gm) | 0.1 (0) \| 0.1 [0, 0.2] | 0.1 (0) \| 0.1 [0, 0.1] | 0.2 (0) \| 0.1 [0.1, 0.2] | 0.000 |
| PFA 20:5 (Eicosapentaenoic) (gm) | 0 (0) \| 0 [0, 0] | 0 (0) \| 0 [0, 0] | 0 (0) \| 0 [0, 0] | 0.077 |
| PFA 22:5 (Docosapentaenoic) (gm) | 0 (0) \| 0 [0, 0] | 0 (0) \| 0 [0, 0] | 0 (0) \| 0 [0, 0] | 0.002 |
| PFA 22:6 (Docosahexaenoic) (gm) | 0 (0) \| 0 [0, 0] | 0 (0) \| 0 [0, 0] | 0 (0) \| 0 [0, 0] | 0.004 |

## Weighted analysis: Laboratory Categorical and Continuous Variables — mec weights

Laboratory characteristics of study participants overall and by sex: Weighted statistics (mean (SD) and median [IQR])).

|  |  |  |  |  |
| --- | --- | --- | --- | --- |
| summary | Overall | Female | Male | p_val |
| Basophils number (1000 cells/uL) | 0 (0) \| 0 [0, 0.1] | 0 (0) \| 0 [0, 0.1] | 0 (0) \| 0 [0, 0.1] | 0.242 |
| Eosinophils number (1000 cells/uL) | 0.2 (0) \| 0.2 [0.1, 0.2] | 0.2 (0) \| 0.1 [0.1, 0.2] | 0.2 (0) \| 0.2 [0.1, 0.3] | 0.000 |
| Lymphocyte number (1000 cells/uL) | 2.3 (0) \| 2.2 [1.9, 2.7] | 2.3 (0) \| 2.2 [1.9, 2.7] | 2.3 (0) \| 2.2 [1.8, 2.6] | 0.216 |
| Monocyte number (1000 cells/uL) | 0.6 (0) \| 0.6 [0.5, 0.7] | 0.6 (0) \| 0.5 [0.4, 0.7] | 0.6 (0) \| 0.6 [0.5, 0.7] | 0.257 |
| Segmented neutrophils num (1000 cell/uL) | 3.9 (0.1) \| 3.6 [2.8, 4.7] | 4.1 (0.1) \| 3.8 [3, 5.2] | 3.8 (0.1) \| 3.5 [2.7, 4.4] | 0.006 |
| Basophils percent (%) | 0.7 (0) \| 0.6 [0.4, 0.8] | 0.6 (0) \| 0.6 [0.4, 0.8] | 0.7 (0) \| 0.6 [0.5, 0.8] | 0.618 |
| Eosinophils percent (%) | 3 (0.1) \| 2.3 [1.5, 3.5] | 2.5 (0.1) \| 2 [1.2, 3] | 3.4 (0.2) \| 2.5 [1.7, 4.3] | 0.000 |
| Hematocrit (%) | 41.4 (0.2) \| 41.2 [39, 43.8] | 39.4 (0.2) \| 39.5 [38, 41.2] | 43.2 (0.2) \| 43.4 [41.2, 45.3] | 0.000 |
| Hydroxycotinine, Serum (ng/mL) | 41.4 (0.2) \| 41.2 [39, 43.8] | 39.4 (0.2) \| 39.5 [38, 41.2] | 43.2 (0.2) \| 43.4 [41.2, 45.3] | 0.000 |
| Hemoglobin (g/dL) | 14 (0.1) \| 13.9 [13.1, 15] | 13.3 (0.1) \| 13.3 [12.7, 13.9] | 14.7 (0.1) \| 14.8 [13.9, 15.5] | 0.000 |
| Lymphocyte percent (%) | 33.9 (0.3) \| 33.9 [28.3, 39.2] | 33.6 (0.4) \| 33 [27.5, 39.2] | 34.2 (0.3) \| 34.7 [28.6, 39.5] | 0.195 |
| Mean cell hemoglobin (pg) | 29.4 (0.1) \| 29.7 [28.4, 30.6] | 29.2 (0.1) \| 29.6 [28.3, 30.5] | 29.5 (0.1) \| 29.7 [28.6, 30.7] | 0.278 |
| Mean cell volume (fL) | 86.6 (0.3) \| 87.1 [84.1, 89.6] | 86.8 (0.3) \| 87.3 [84.2, 90.3] | 86.5 (0.4) \| 86.8 [84.1, 89.1] | 0.075 |
| Monocyte percent (%) | 8.4 (0.1) \| 8.1 [6.8, 9.5] | 8.1 (0.2) \| 7.9 [6.5, 9.1] | 8.6 (0.1) \| 8.4 [7.1, 9.9] | 0.000 |
| Mean platelet volume (fL) | 8.4 (0.1) \| 8.4 [7.8, 8.9] | 8.5 (0.1) \| 8.5 [7.9, 9] | 8.3 (0) \| 8.2 [7.8, 8.8] | 0.009 |
| Segmented neutrophils percent (%) | 54.1 (0.4) \| 53.8 [47.6, 60.4] | 55.3 (0.6) \| 55 [48.1, 62.2] | 53.1 (0.4) \| 52.8 [47, 58.8] | 0.003 |
| Platelet count (1000 cells/uL) | 250.3 (1.3) \| 246 [215, 282] | 258.5 (1.8) \| 255 [226, 285] | 242.8 (2.3) \| 242 [207, 273] | 0.001 |
| Red blood cell count (million cells/uL) | 4.8 (0) \| 4.7 [4.5, 5.1] | 4.6 (0) \| 4.6 [4.3, 4.7] | 5 (0) \| 5 [4.7, 5.2] | 0.000 |
| Red cell distribution width (%) | 13.3 (0) \| 13.1 [12.7, 13.7] | 13.3 (0.1) \| 13.1 [12.6, 13.7] | 13.3 (0) \| 13.2 [12.8, 13.7] | 0.010 |
| White blood cell count (1000 cells/uL) | 7.1 (0.1) \| 6.9 [5.6, 8.1] | 7.3 (0.1) \| 7.1 [5.9, 8.4] | 6.9 (0.1) \| 6.6 [5.4, 7.9] | 0.022 |
| Albumin (g/L) | 44.8 (0.1) \| 45 [43, 47] | 44.2 (0.1) \| 44 [43, 46] | 45.4 (0.2) \| 46 [44, 47] | 0.000 |
| Blood urea nitrogen (mmol/L) | 3.8 (0.1) \| 3.6 [2.9, 4.6] | 3.7 (0.1) \| 3.6 [2.9, 4.3] | 4 (0.1) \| 3.9 [3.2, 4.6] | 0.001 |
| Total calcium (mmol/L) | 2.4 (0) \| 2.4 [2.4, 2.5] | 2.4 (0) \| 2.4 [2.4, 2.5] | 2.4 (0) \| 2.4 [2.4, 2.5] | 0.002 |
| Cholesterol (mmol/L) | 4.1 (0) \| 4 [3.5, 4.5] | 4.2 (0.1) \| 4.1 [3.7, 4.6] | 3.9 (0) \| 3.9 [3.4, 4.4] | 0.001 |
| Globulin (g/L) | 26.9 (0.3) \| 27 [24, 29] | 27.6 (0.3) \| 27 [25, 30] | 26.4 (0.3) \| 26 [24, 28] | 0.000 |
| Glucose, refrigerated serum (mmol/L) | 5 (0) \| 4.9 [4.7, 5.3] | 5 (0) \| 4.9 [4.7, 5.2] | 5 (0) \| 5 [4.7, 5.3] | 0.012 |
| Iron, refrigerated serum (umol/L) | 15 (0.2) \| 14.1 [10.6, 19] | 14 (0.4) \| 12.9 [9.5, 18.3] | 15.9 (0.4) \| 15.2 [11.8, 19.3] | 0.006 |
| Phosphorus (mmol/L) | 1.4 (0) \| 1.4 [1.3, 1.6] | 1.4 (0) \| 1.4 [1.3, 1.5] | 1.5 (0) \| 1.5 [1.3, 1.6] | 0.000 |
| Total bilirubin (umol/L) | 11.1 (0.2) \| 10.3 [6.8, 13.7] | 10.2 (0.3) \| 8.6 [6.8, 12] | 12 (0.4) \| 10.3 [8.6, 13.7] | 0.002 |
| Total protein (g/L) | 71.7 (0.3) \| 72 [69, 74] | 71.8 (0.3) \| 72 [69, 75] | 71.7 (0.3) \| 72 [69, 74] | 0.886 |
| Triglycerides, refrigerated (mmol/L) | 1.1 (0) \| 0.9 [0.6, 1.3] | 1.1 (0.1) \| 0.8 [0.6, 1.2] | 1.2 (0.1) \| 0.9 [0.6, 1.4] | 0.007 |
| Uric acid (umol/L) | 301.5 (3.3) \| 297.4 [243.9, 350.9] | 269.6 (5.5) \| 267.7 [226, 309.3] | 331.2 (4.2) \| 327.1 [285.5, 374.7] | 0.000 |
| Alkaline phosphatase (IU/L) | 138.3 (3.5) \| 100 [70, 188] | 97.7 (2.9) \| 79 [59, 112] | 176 (4.8) \| 148 [92, 239] | 0.000 |
| Aspartate aminotransferase AST (IU/L) | 23.3 (0.3) \| 22 [19, 26] | 21.7 (0.5) \| 20 [18, 23] | 24.8 (0.3) \| 24 [21, 27] | 0.000 |
| Alanine aminotransferase ALT (IU/L) | 19.6 (0.4) \| 16 [14, 22] | 17.3 (0.6) \| 15 [13, 18] | 21.7 (0.6) \| 19 [15, 24] | 0.000 |
| Bicarbonate (mmol/L) | 24.5 (0.2) \| 25 [23, 26] | 23.8 (0.2) \| 24 [23, 25] | 25.2 (0.2) \| 25 [24, 26] | 0.000 |
| Chloride (mmol/L) | 104.8 (0.2) \| 105 [103, 106] | 105.5 (0.2) \| 105 [104, 107] | 104.2 (0.2) \| 104 [103, 105] | 0.000 |
| Creatinine (mg/dL) | 0.7 (0) \| 0.7 [0.6, 0.8] | 0.7 (0) \| 0.7 [0.6, 0.8] | 0.8 (0) \| 0.8 [0.7, 0.9] | 0.000 |
| Gamma glutamyl transferase (U/L) | 14 (0.3) \| 12 [10, 16] | 12.7 (0.4) \| 11 [9, 14] | 15.3 (0.4) \| 13 [11, 17] | 0.000 |
| Potassium (mmol/L) | 4.1 (0) \| 4 [3.8, 4.3] | 4 (0) \| 4 [3.8, 4.2] | 4.1 (0) \| 4.1 [3.9, 4.3] | 0.004 |
| Lactate dehydrogenase (U/L) | 129.2 (1.5) \| 126 [110, 143] | 123 (1.7) \| 122 [106, 134] | 134.9 (1.6) \| 132 [116, 153] | 0.000 |
| Sodium (mmol/L) | 139.9 (0.1) \| 140 [139, 141] | 139.8 (0.1) \| 140 [139, 141] | 140.1 (0.1) \| 140 [139, 141] | 0.044 |
| Osmolality (mmol/Kg) | 278.1 (0.2) \| 278 [276, 280] | 277.7 (0.3) \| 277 [275, 280] | 278.5 (0.2) \| 279 [276, 281] | 0.002 |
| Albumin, urine (mg/L) | 34.3 (3.6) \| 9.9 [5.3, 21] | 36.5 (4.3) \| 13.5 [5.8, 27.2] | 32.3 (6.5) \| 8.6 [4.9, 15.4] | 0.000 |
| Cotinine, Serum (ng/mL) | 11.2 (2.2) \| 0 [0, 0.2] | 7.8 (3.1) \| 0 [0, 0.1] | 14.4 (3.6) \| 0 [0, 0.5] | 0.075 |
| Glycohemoglobin (%) | 5.2 (0) \| 5.2 [5, 5.4] | 5.2 (0) \| 5.2 [5, 5.4] | 5.2 (0) \| 5.2 [5, 5.4] | 0.251 |
| BMI CDC normalization | 0.7 (0.1) \| 0.7 [0, 1.6] | 0.7 (0.1) \| 0.7 [0, 1.6] | 0.7 (0.1) \| 0.7 [-0.1, 1.6] | 0.923 |
| BMI CDC normalization percentage | 0.7 (0) \| 0.8 [0.5, 0.9] | 0.7 (0) \| 0.8 [0.5, 0.9] | 0.7 (0) \| 0.8 [0.5, 0.9] | 0.909 |

Physical activity and BMI (cat.): Weighted statistics (percentage [95% CI])).

|  |  |  |  |  |  |
| --- | --- | --- | --- | --- | --- |
| summary | values | Overall | Female | Male | p_val |
| Physical activity | No | 39 [34.8%, 43.2%] | 48.5 [42.5%, 54.6%] | 30.1 [26.5%, 33.8%] | 0.000 |
| Physical activity | Yes | 61 [56.8%, 65.2%] | 51.5 [45.4%, 57.5%] | 69.9 [66.2%, 73.5%] |  |
| BMI categories | Underweight | 2.4 [1.1%, 3.8%] | 1.8 [0.7%, 3%] | 3 [0.9%, 5.1%] | 0.634 |
| BMI categories | Healthy Weight | 58.5 [53.8%, 63.2%] | 59.4 [51.3%, 67.4%] | 57.7 [52.9%, 62.5%] |  |
| BMI categories | Overweight | 18.5 [15.5%, 21.5%] | 17.6 [13.8%, 21.4%] | 19.3 [14.9%, 23.7%] |  |
| BMI categories | Obese | 20.6 [16.2%, 24.9%] | 21.2 [14.3%, 28%] | 20 [15%, 25.1%] |  |

## QQ-plots


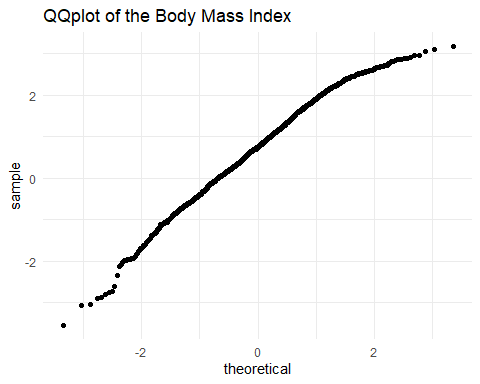


# Session Information

sessionInfo()

## R version 4.1.2 (2021-11-01)
## Platform: x86_64-w64-mingw32/x64 (64-bit)
## Running under: Windows 10 x64 (build 19044)
##
## Matrix products: default
##
## locale:
## [1] LC_COLLATE=English_United States.1252 LC_CTYPE=English_United States.1252 LC_MONETARY=English_United States.1252
## [4] LC_NUMERIC=C LC_TIME=English_United States.1252
##
## attached base packages:
## [1] grid stats graphics grDevices utils datasets methods base
##
## other attached packages:
## [1] knitr_1.37 broom_0.7.10 tableone_0.13.0 survey_4.1-1 Matrix_1.3-4 SASxport_1.7.0
## [7] childsds_0.7.6 srvyr_1.1.0 flextable_0.6.10 DT_0.20 Hmisc_4.6-0 Formula_1.2-4
## [13] survival_3.2-13 lattice_0.20-45 data.table_1.14.2 tictoc_1.0.1 XML_3.99-0.8 janitor_2.1.0
## [19] forcats_0.5.1 stringr_1.4.0 dplyr_1.0.7 purrr_0.3.4 readr_2.1.1 tidyr_1.1.4
## [25] tibble_3.1.6 ggplot2_3.3.5 tidyverse_1.3.1 haven_2.4.3
##
## loaded via a namespace (and not attached):
## [1] TH.data_1.1-0 minqa_1.2.4 colorspace_2.0-2 class_7.3-19 ellipsis_0.3.2
## [6] sjlabelled_1.1.8 estimability_1.3 snakecase_0.11.0 htmlTable_2.3.0 parameters_0.15.0
## [11] base64enc_0.1-3 fs_1.5.2 proxy_0.4-26 rstudioapi_0.13 farver_2.1.0
## [16] ggfittext_0.9.1 fansi_0.5.0 mvtnorm_1.1-3 lubridate_1.8.0 xml2_1.3.3
## [21] codetools_0.2-18 splines_4.1.2 sjmisc_2.8.9 jsonlite_1.7.2 nloptr_1.2.2.3
## [26] ggeffects_1.1.1 cluster_2.1.2 dbplyr_2.1.1 png_0.1-7 gamlss.dist_6.0-1
## [31] effectsize_0.5 inspectdf_0.0.11 compiler_4.1.2 httr_1.4.2 sjstats_0.18.1
## [36] emmeans_1.7.1-1 backports_1.4.1 assertthat_0.2.1 fastmap_1.1.0 cli_3.1.0
## [41] prettyunits_1.1.1 htmltools_0.5.2 tools_4.1.2 coda_0.19-4 gtable_0.3.0
## [46] glue_1.6.0 Rcpp_1.0.7 cellranger_1.1.0 vctrs_0.3.8 sjPlot_2.8.10
## [51] nlme_3.1-153 insight_0.14.5 xfun_0.29 lme4_1.1-27.1 rvest_1.0.2
## [56] lifecycle_1.0.1 MASS_7.3-54 zoo_1.8-9 scales_1.1.1 hms_1.1.1
## [61] sandwich_3.0-1 RColorBrewer_1.1-2 yaml_2.2.1 gridExtra_2.3 gdtools_0.2.3
## [66] rpart_4.1-15 latticeExtra_0.6-29 stringi_1.7.6 highr_0.9 bayestestR_0.11.5
## [71] e1071_1.7-9 checkmate_2.0.0 zip_2.2.0 boot_1.3-28 rlang_0.4.12
## [76] pkgconfig_2.0.3 systemfonts_1.0.3 evaluate_0.14 labeling_0.4.2 htmlwidgets_1.5.4
## [81] tidyselect_1.1.1 namer_0.1.5 plyr_1.8.6 magrittr_2.0.1 R6_2.5.1
## [86] generics_0.1.1 multcomp_1.4-17 DBI_1.1.2 pillar_1.6.4 foreign_0.8-81
## [91] withr_2.4.3 datawizard_0.2.1 nnet_7.3-16 performance_0.8.0 modelr_0.1.8
## [96] crayon_1.4.2 uuid_1.0-3 utf8_1.2.2 officer_0.4.1 tzdb_0.2.0
## [101] rmarkdown_2.11 progress_1.2.2 jpeg_0.1-9 readxl_1.3.1 reprex_2.0.1
## [106] digest_0.6.29 xtable_1.8-4 munsell_0.5.0 mitools_2.4

## References

| x |
| --- |
| David Gohel (2021). flextable: Functions for Tabular Reporting. R package version 0.6.10. <https://CRAN.R-project.org/package=flextable> |
| David Robinson, Alex Hayes and Simon Couch (2021). broom: Convert Statistical Objects into Tidy Tibbles. R package version 0.7.10. <https://CRAN.R-project.org/package=broom> |
| Douglas Bates and Martin Maechler (2021). Matrix: Sparse and Dense Matrix Classes and Methods. R package version 1.3-4. <https://CRAN.R-project.org/package=Matrix> |
| Duncan Temple Lang (2021). XML: Tools for Parsing and Generating XML Within R and S-Plus. R package version 3.99-0.8. <https://CRAN.R-project.org/package=XML> |
| Frank E Harrell Jr (2021). Hmisc: Harrell Miscellaneous. R package version 4.6-0. <https://CRAN.R-project.org/package=Hmisc> |
| Greg Freedman Ellis and Ben Schneider (2021). srvyr: ‘dplyr’-Like Syntax for Summary Statistics of Survey Data. R package version 1.1.0. <https://CRAN.R-project.org/package=srvyr> |
| Gregory R. Warnes – Unless otherwise noted, the contents of this package were written by Gregory R. Warnes, are provided under the terms of the GNU General Public License, version 2.0 or later. – The files ‘src/ieee2ibm.c’, ‘src/ibm2ieee.c’ were extracted from BRL-CAD file /brlcad/src/libbu/htond.c written by Michael John Muuss, Copyright 2004-2007 United States Government as represented by the U.S. Army Research Laboratory, is utilized, redistributed under the terms of the GNU Lesser General Public License, version 2.1. – The file ‘R/read.xport.R’ is adapted from the ‘Hmisc’ package created by Frank E. Harrell, Jr., is utilized, redistributed under the terms of the GNU General Public License, version 2.0 or later. – The files ‘R/xport.R’, ‘src/SASxport.c’, ‘src/SASxport.h’, ‘src/foreign.h’ are copied or adapted from the ‘R’ ‘foreign’ package created by Douglas M. Bates, Saikat DebRoy, are utilized, redistributed under the terms of the GNU General Public License and version 2.0 or later. – The creation of this package was partially funded by Metrum Institute. (2020). SASxport: Read and Write ‘SAS’ ‘XPORT’ Files. R package version 1.7.0. <https://CRAN.R-project.org/package=SASxport> |
| H. Wickham. ggplot2: Elegant Graphics for Data Analysis. Springer-Verlag New York, 2016. |
| Hadley Wickham (2019). stringr: Simple, Consistent Wrappers for Common String Operations. R package version 1.4.0. <https://CRAN.R-project.org/package=stringr> |
| Hadley Wickham (2021). forcats: Tools for Working with Categorical Variables (Factors). R package version 0.5.1. <https://CRAN.R-project.org/package=forcats> |
| Hadley Wickham (2021). tidyr: Tidy Messy Data. R package version 1.1.4. <https://CRAN.R-project.org/package=tidyr> |
| Hadley Wickham and Evan Miller (2021). haven: Import and Export ‘SPSS’, ‘Stata’ and ‘SAS’ Files. R package version 2.4.3. <https://CRAN.R-project.org/package=haven> |
| Hadley Wickham, Jim Hester and Jennifer Bryan (2021). readr: Read Rectangular Text Data. R package version 2.1.1. <https://CRAN.R-project.org/package=readr> |
| Hadley Wickham, Romain François, Lionel Henry and Kirill Müller (2021). dplyr: A Grammar of Data Manipulation. R package version 1.0.7. <https://CRAN.R-project.org/package=dplyr> |
| Kazuki Yoshida and Alexander Bartel (2021). tableone: Create ‘Table 1’ to Describe Baseline Characteristics with or without Propensity Score Weights. R package version 0.13.0. <https://CRAN.R-project.org/package=tableone> |
| Kirill Müller and Hadley Wickham (2021). tibble: Simple Data Frames. R package version 3.1.6. <https://CRAN.R-project.org/package=tibble> |
| Lionel Henry and Hadley Wickham (2020). purrr: Functional Programming Tools. R package version 0.3.4. <https://CRAN.R-project.org/package=purrr> |
| Mandy Vogel (2020). childsds: Data and Methods Around Reference Values in Pediatrics. R package version 0.7.6. <https://CRAN.R-project.org/package=childsds> |
| Matt Dowle and Arun Srinivasan (2021). data.table: Extension of data.frame. R package version 1.14.2. <https://CRAN.R-project.org/package=data.table> |
| R Core Team (2021). R: A language and environment for statistical computing. R Foundation for Statistical Computing, Vienna, Austria. URL <https://www.R-project.org/>. |
| Sam Firke (2021). janitor: Simple Tools for Examining and Cleaning Dirty Data. R package version 2.1.0. <https://CRAN.R-project.org/package=janitor> |
| Sarkar, Deepayan (2008) Lattice: Multivariate Data Visualization with R. Springer, New York. ISBN 978-0-387-75968-5 |
| Sergei Izrailev (2021). tictoc: Functions for Timing R Scripts, as Well as Implementations of Stack and List Structures. R package version 1.0.1. <https://CRAN.R-project.org/package=tictoc> |
| T. Lumley (2020) “survey: analysis of complex survey samples”. R package version 4.0. |
| Therneau T (2021). *A Package for Survival Analysis in R*. R package version 3.2-13, <URL:<https://CRAN.R-project.org/package=survival>>. |
| Wickham et al., (2019). Welcome to the tidyverse. Journal of Open Source Software, 4(43), 1686, <https://doi.org/10.21105/joss.01686> |
| Yihui Xie (2021). knitr: A General-Purpose Package for Dynamic Report Generation in R. R package version 1.37. |
| Yihui Xie, Joe Cheng and Xianying Tan (2021). DT: A Wrapper of the JavaScript Library ‘DataTables’. R package version 0.20. <https://CRAN.R-project.org/package=DT> |
| Zeileis A, Croissant Y (2010). “Extended Model Formulas in R: Multiple Parts and Multiple Responses.” *Journalof Statistical Software*, *34*(1), 1-13. doi: 10.18637/jss.v034.i01 (URL:<https://doi.org/10.18637/jss.v034.i01>). |
