# Supplementary material for: An exposome-wide association study on body mass index in adolescents using the National Health and Nutrition Examination Survey (NHANES) 2003–2004 and 2013–2014 data
Source: Sci Rep. 2022 May 25;12:8856. doi: 10.1038/s41598-022-12459-z (PMC9132896; doi:10.1038/s41598-022-12459-z)
Supplement: Supplementary file 1 — Supplementary Information. [file 41598_2022_12459_MOESM1_ESM.zip › SupplementaryMaterial_DataAnalysisDetails - Copy/wordoutput/S3_SensitivityAnalysis.docx]

Environment-wide association study on body mass index of 12-18 year-olds, US NHANES 2003-2004 and 2013-2014

Water and Health Laboratory - Cyprus University of Technology

# Sensitivity Analysis: non-obese target study participants for 2003-2004 dataset

## Univariate regressions non-obese target study participants 2003-2004

Univariate regression results for the non-obese study participants - FDR - BH method <0.05

| variable | term | estimate | std.error | statistic | p.value | Category | fdr | var_name | summary |
| --- | --- | --- | --- | --- | --- | --- | --- | --- | --- |

## Multivariable regressions non-obese target study participants 2003-2004

Multivariable regression results for the non-obese study participants - FDR - BH method <0.05

| variable | term | estimate | std.error | statistic | p.value | Category | fdr | var_name | summary |
| --- | --- | --- | --- | --- | --- | --- | --- | --- | --- |

# Sensitivity analysis in replication dataset: 2013-2014

## Univariate regressions non-obese target study participants 2013-2014

Univariate regression results for the non-obese study participants - FDR<0.05

| summary | Category | estimate | std.error | statistic | p.value | fdr |
| --- | --- | --- | --- | --- | --- | --- |
| Vitamin B12 (mcg) | Nutrition (day1) | -0.078 | 0.016 | -4.825 | 0.000 | 0.015 |
| Gamma glutamyl transferase (U/L) | Laboratory | 0.173 | 0.038 | 4.547 | 0.000 | 0.015 |
| Albumin (g/L) | Laboratory | -0.111 | 0.025 | -4.532 | 0.000 | 0.015 |
| Alanine aminotransferase ALT (IU/L) | Laboratory | 0.177 | 0.040 | 4.391 | 0.001 | 0.015 |
| Triglycerides, refrigerated (mmol/L) | Laboratory | 0.180 | 0.041 | 4.343 | 0.001 | 0.015 |
| Folic acid (mcg) | Nutrition (day1) | -0.091 | 0.023 | -3.902 | 0.002 | 0.026 |
| Cholesterol (mg) | Nutrition (day1) | -0.057 | 0.015 | -3.822 | 0.002 | 0.026 |
| Blood urea nitrogen (mmol/L) | Laboratory | 0.096 | 0.025 | 3.813 | 0.002 | 0.026 |
| White blood cell count (1000 cells/uL) | Laboratory | 0.110 | 0.031 | 3.545 | 0.003 | 0.039 |

## Multivariable regressions non-obese target study participants 2013-2014

Multivariable regression results for the non-obese study participants - FDR<0.05

| variable | term | estimate | std.error | statistic | p.value | Category | fdr | var_name | summary |
| --- | --- | --- | --- | --- | --- | --- | --- | --- | --- |

# Session Information

sessionInfo()

## R version 4.1.2 (2021-11-01)
## Platform: x86_64-w64-mingw32/x64 (64-bit)
## Running under: Windows 10 x64 (build 19044)
##
## Matrix products: default
##
## locale:
## [1] LC_COLLATE=English_United States.1252 LC_CTYPE=English_United States.1252 LC_MONETARY=English_United States.1252
## [4] LC_NUMERIC=C LC_TIME=English_United States.1252
##
## attached base packages:
## [1] grid stats graphics grDevices utils datasets methods base
##
## other attached packages:
## [1] corrplot_0.92 patchwork_1.1.1 jtools_2.1.4 knitr_1.37 broom_0.7.10 tableone_0.13.0
## [7] survey_4.1-1 Matrix_1.3-4 SASxport_1.7.0 childsds_0.7.6 srvyr_1.1.0 flextable_0.6.10
## [13] DT_0.20 Hmisc_4.6-0 Formula_1.2-4 survival_3.2-13 lattice_0.20-45 data.table_1.14.2
## [19] tictoc_1.0.1 XML_3.99-0.8 janitor_2.1.0 forcats_0.5.1 stringr_1.4.0 dplyr_1.0.7
## [25] purrr_0.3.4 readr_2.1.1 tidyr_1.1.4 tibble_3.1.6 ggplot2_3.3.5 tidyverse_1.3.1
## [31] haven_2.4.3
##
## loaded via a namespace (and not attached):
## [1] readxl_1.3.1 uuid_1.0-3 backports_1.4.1 systemfonts_1.0.3 plyr_1.8.6 splines_4.1.2
## [7] TH.data_1.1-0 digest_0.6.29 htmltools_0.5.2 fansi_0.5.0 magrittr_2.0.1 checkmate_2.0.0
## [13] cluster_2.1.2 tzdb_0.2.0 ggfittext_0.9.1 modelr_0.1.8 vroom_1.5.7 officer_0.4.1
## [19] sandwich_3.0-1 prettyunits_1.1.1 jpeg_0.1-9 colorspace_2.0-2 rvest_1.0.2 mitools_2.4
## [25] textshaping_0.3.6 xfun_0.29 crayon_1.4.2 jsonlite_1.7.2 lme4_1.1-27.1 zoo_1.8-9
## [31] glue_1.6.0 gtable_0.3.0 emmeans_1.7.1-1 sjstats_0.18.1 sjmisc_2.8.9 scales_1.1.1
## [37] mvtnorm_1.1-3 DBI_1.1.2 ggeffects_1.1.1 Rcpp_1.0.7 xtable_1.8-4 progress_1.2.2
## [43] performance_0.8.0 htmlTable_2.3.0 bit_4.0.4 foreign_0.8-81 proxy_0.4-26 datawizard_0.2.1
## [49] htmlwidgets_1.5.4 httr_1.4.2 RColorBrewer_1.1-2 ellipsis_0.3.2 pkgconfig_2.0.3 farver_2.1.0
## [55] nnet_7.3-16 dbplyr_2.1.1 utf8_1.2.2 tidyselect_1.1.1 labeling_0.4.2 rlang_0.4.12
## [61] effectsize_0.5 munsell_0.5.0 cellranger_1.1.0 tools_4.1.2 cli_3.1.0 generics_0.1.1
## [67] sjlabelled_1.1.8 evaluate_0.14 fastmap_1.1.0 ragg_1.2.1 yaml_2.2.1 bit64_4.0.5
## [73] fs_1.5.2 namer_0.1.5 pander_0.6.4 zip_2.2.0 nlme_3.1-153 report_0.4.0
## [79] xml2_1.3.3 compiler_4.1.2 rstudioapi_0.13 png_0.1-7 e1071_1.7-9 reprex_2.0.1
## [85] stringi_1.7.6 highr_0.9 parameters_0.15.0 gdtools_0.2.3 nloptr_1.2.2.3 vctrs_0.3.8
## [91] pillar_1.6.4 lifecycle_1.0.1 inspectdf_0.0.11 estimability_1.3 insight_0.14.5 R6_2.5.1
## [97] latticeExtra_0.6-29 gridExtra_2.3 codetools_0.2-18 boot_1.3-28 MASS_7.3-54 assertthat_0.2.1
## [103] withr_2.4.3 multcomp_1.4-17 parallel_4.1.2 bayestestR_0.11.5 hms_1.1.1 rpart_4.1-15
## [109] sjPlot_2.8.10 coda_0.19-4 class_7.3-19 minqa_1.2.4 rmarkdown_2.11 snakecase_0.11.0
## [115] gamlss.dist_6.0-1 lubridate_1.8.0 base64enc_0.1-3

| x |
| --- |
| David Gohel (2021). flextable: Functions for Tabular Reporting. R package version 0.6.10. <https://CRAN.R-project.org/package=flextable> |
| David Robinson, Alex Hayes and Simon Couch (2021). broom: Convert Statistical Objects into Tidy Tibbles. R package version 0.7.10. <https://CRAN.R-project.org/package=broom> |
| Douglas Bates and Martin Maechler (2021). Matrix: Sparse and Dense Matrix Classes and Methods. R package version 1.3-4. <https://CRAN.R-project.org/package=Matrix> |
| Duncan Temple Lang (2021). XML: Tools for Parsing and Generating XML Within R and S-Plus. R package version 3.99-0.8. <https://CRAN.R-project.org/package=XML> |
| Frank E Harrell Jr (2021). Hmisc: Harrell Miscellaneous. R package version 4.6-0. <https://CRAN.R-project.org/package=Hmisc> |
| Greg Freedman Ellis and Ben Schneider (2021). srvyr: ‘dplyr’-Like Syntax for Summary Statistics of Survey Data. R package version 1.1.0. <https://CRAN.R-project.org/package=srvyr> |
| Gregory R. Warnes – Unless otherwise noted, the contents of this package were written by Gregory R. Warnes, are provided under the terms of the GNU General Public License, version 2.0 or later. – The files ‘src/ieee2ibm.c’, ‘src/ibm2ieee.c’ were extracted from BRL-CAD file /brlcad/src/libbu/htond.c written by Michael John Muuss, Copyright 2004-2007 United States Government as represented by the U.S. Army Research Laboratory, is utilized, redistributed under the terms of the GNU Lesser General Public License, version 2.1. – The file ‘R/read.xport.R’ is adapted from the ‘Hmisc’ package created by Frank E. Harrell, Jr., is utilized, redistributed under the terms of the GNU General Public License, version 2.0 or later. – The files ‘R/xport.R’, ‘src/SASxport.c’, ‘src/SASxport.h’, ‘src/foreign.h’ are copied or adapted from the ‘R’ ‘foreign’ package created by Douglas M. Bates, Saikat DebRoy, are utilized, redistributed under the terms of the GNU General Public License and version 2.0 or later. – The creation of this package was partially funded by Metrum Institute. (2020). SASxport: Read and Write ‘SAS’ ‘XPORT’ Files. R package version 1.7.0. <https://CRAN.R-project.org/package=SASxport> |
| H. Wickham. ggplot2: Elegant Graphics for Data Analysis. Springer-Verlag New York, 2016. |
| Hadley Wickham (2019). stringr: Simple, Consistent Wrappers for Common String Operations. R package version 1.4.0. <https://CRAN.R-project.org/package=stringr> |
| Hadley Wickham (2021). forcats: Tools for Working with Categorical Variables (Factors). R package version 0.5.1. <https://CRAN.R-project.org/package=forcats> |
| Hadley Wickham (2021). tidyr: Tidy Messy Data. R package version 1.1.4. <https://CRAN.R-project.org/package=tidyr> |
| Hadley Wickham and Evan Miller (2021). haven: Import and Export ‘SPSS’, ‘Stata’ and ‘SAS’ Files. R package version 2.4.3. <https://CRAN.R-project.org/package=haven> |
| Hadley Wickham, Jim Hester and Jennifer Bryan (2021). readr: Read Rectangular Text Data. R package version 2.1.1. <https://CRAN.R-project.org/package=readr> |
| Hadley Wickham, Romain François, Lionel Henry and Kirill Müller (2021). dplyr: A Grammar of Data Manipulation. R package version 1.0.7. <https://CRAN.R-project.org/package=dplyr> |
| Kazuki Yoshida and Alexander Bartel (2021). tableone: Create ‘Table 1’ to Describe Baseline Characteristics with or without Propensity Score Weights. R package version 0.13.0. <https://CRAN.R-project.org/package=tableone> |
| Kirill Müller and Hadley Wickham (2021). tibble: Simple Data Frames. R package version 3.1.6. <https://CRAN.R-project.org/package=tibble> |
| Lionel Henry and Hadley Wickham (2020). purrr: Functional Programming Tools. R package version 0.3.4. <https://CRAN.R-project.org/package=purrr> |
| Long JA (2020). *jtools: Analysis and Presentation of Social Scientific Data*. R package version 2.1.0, <URL:<https://cran.r-project.org/package=jtools>>. |
| Mandy Vogel (2020). childsds: Data and Methods Around Reference Values in Pediatrics. R package version 0.7.6. <https://CRAN.R-project.org/package=childsds> |
| Matt Dowle and Arun Srinivasan (2021). data.table: Extension of data.frame. R package version 1.14.2. <https://CRAN.R-project.org/package=data.table> |
| R Core Team (2021). R: A language and environment for statistical computing. R Foundation for Statistical Computing, Vienna, Austria. URL <https://www.R-project.org/>. |
| Sam Firke (2021). janitor: Simple Tools for Examining and Cleaning Dirty Data. R package version 2.1.0. <https://CRAN.R-project.org/package=janitor> |
| Sarkar, Deepayan (2008) Lattice: Multivariate Data Visualization with R. Springer, New York. ISBN 978-0-387-75968-5 |
| Sergei Izrailev (2021). tictoc: Functions for Timing R Scripts, as Well as Implementations of Stack and List Structures. R package version 1.0.1. <https://CRAN.R-project.org/package=tictoc> |
| T. Lumley (2020) “survey: analysis of complex survey samples”. R package version 4.0. |
| Taiyun Wei and Viliam Simko (2021). R package ‘corrplot’: Visualization of a Correlation Matrix (Version 0.92). Available from <https://github.com/taiyun/corrplot> |
| Therneau T (2021). *A Package for Survival Analysis in R*. R package version 3.2-13, <URL:<https://CRAN.R-project.org/package=survival>>. |
| Thomas Lin Pedersen (2020). patchwork: The Composer of Plots. R package version 1.1.1. <https://CRAN.R-project.org/package=patchwork> |
| Wickham et al., (2019). Welcome to the tidyverse. Journal of Open Source Software, 4(43), 1686, <https://doi.org/10.21105/joss.01686> |
| Yihui Xie (2021). knitr: A General-Purpose Package for Dynamic Report Generation in R. R package version 1.37. |
| Yihui Xie, Joe Cheng and Xianying Tan (2021). DT: A Wrapper of the JavaScript Library ‘DataTables’. R package version 0.20. <https://CRAN.R-project.org/package=DT> |
| Zeileis A, Croissant Y (2010). “Extended Model Formulas in R: Multiple Parts and Multiple Responses.” *Journalof Statistical Software*, *34*(1), 1-13. doi: 10.18637/jss.v034.i01 (URL:<https://doi.org/10.18637/jss.v034.i01>). |
