# Supplementary figures and images for: An exposome-wide association study on body mass index in adolescents using the National Health and Nutrition Examination Survey (NHANES) 2003–2004 and 2013–2014 data
Source: Sci Rep. 2022 May 25;12:8856. doi: 10.1038/s41598-022-12459-z (PMC9132896; doi:10.1038/s41598-022-12459-z)

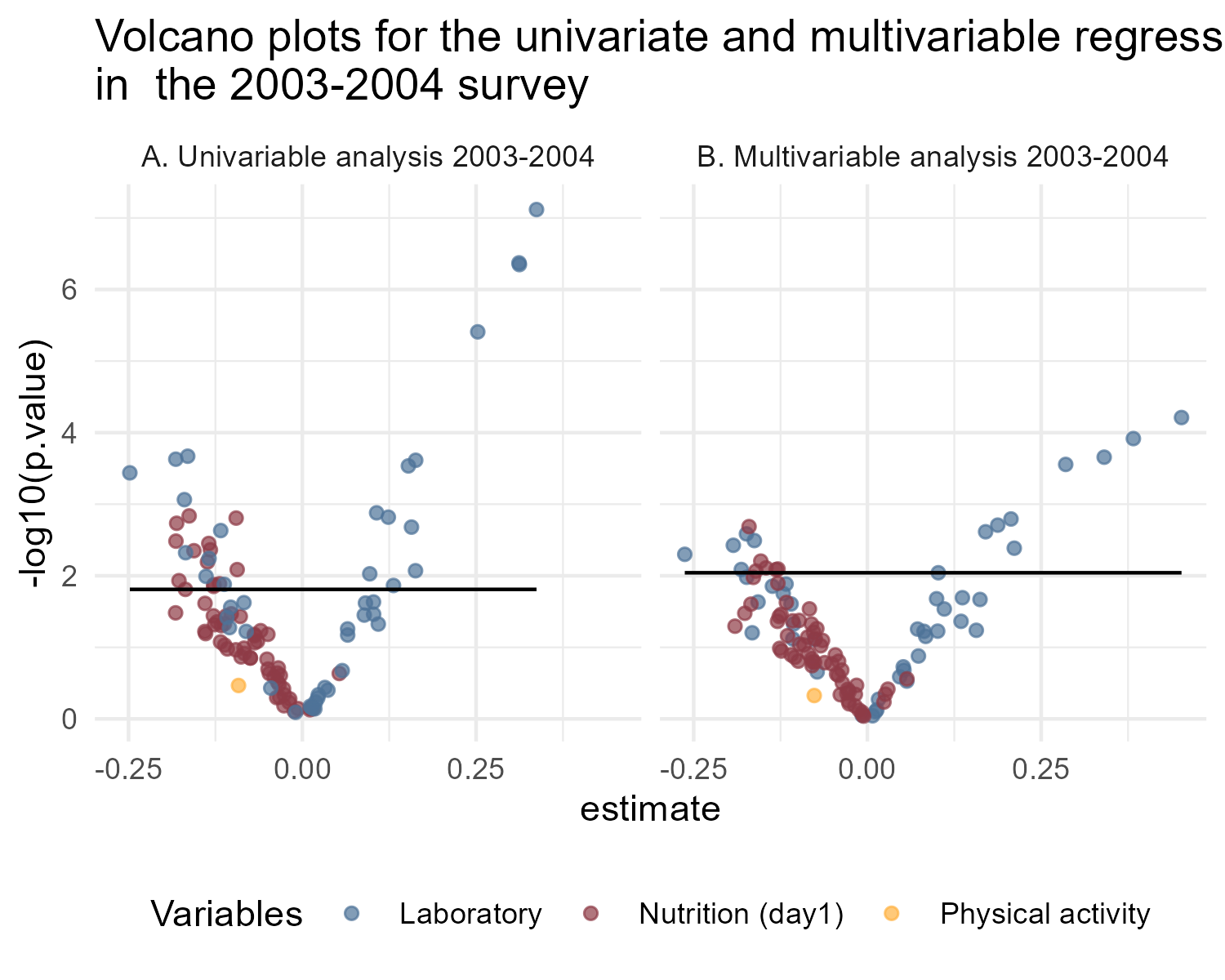

Supplement: Supplementary file 1 — Supplementary Information. [file 41598_2022_12459_MOESM1_ESM.zip › SupplementaryMaterial_DataAnalysisDetails - Copy/figures/figure3.png]

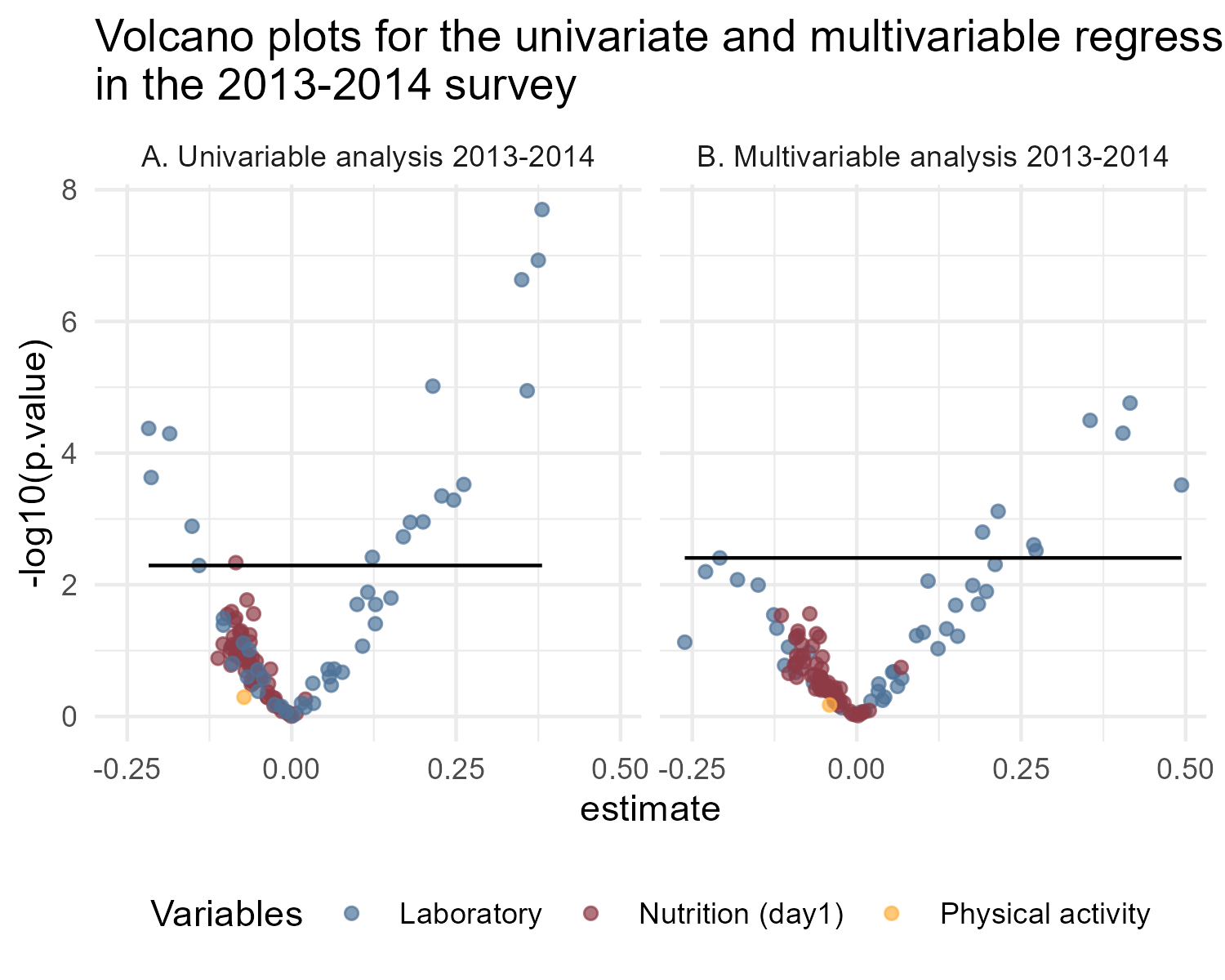

Supplement: Supplementary file 1 — Supplementary Information. [file 41598_2022_12459_MOESM1_ESM.zip › SupplementaryMaterial_DataAnalysisDetails - Copy/figures/figure4.png]

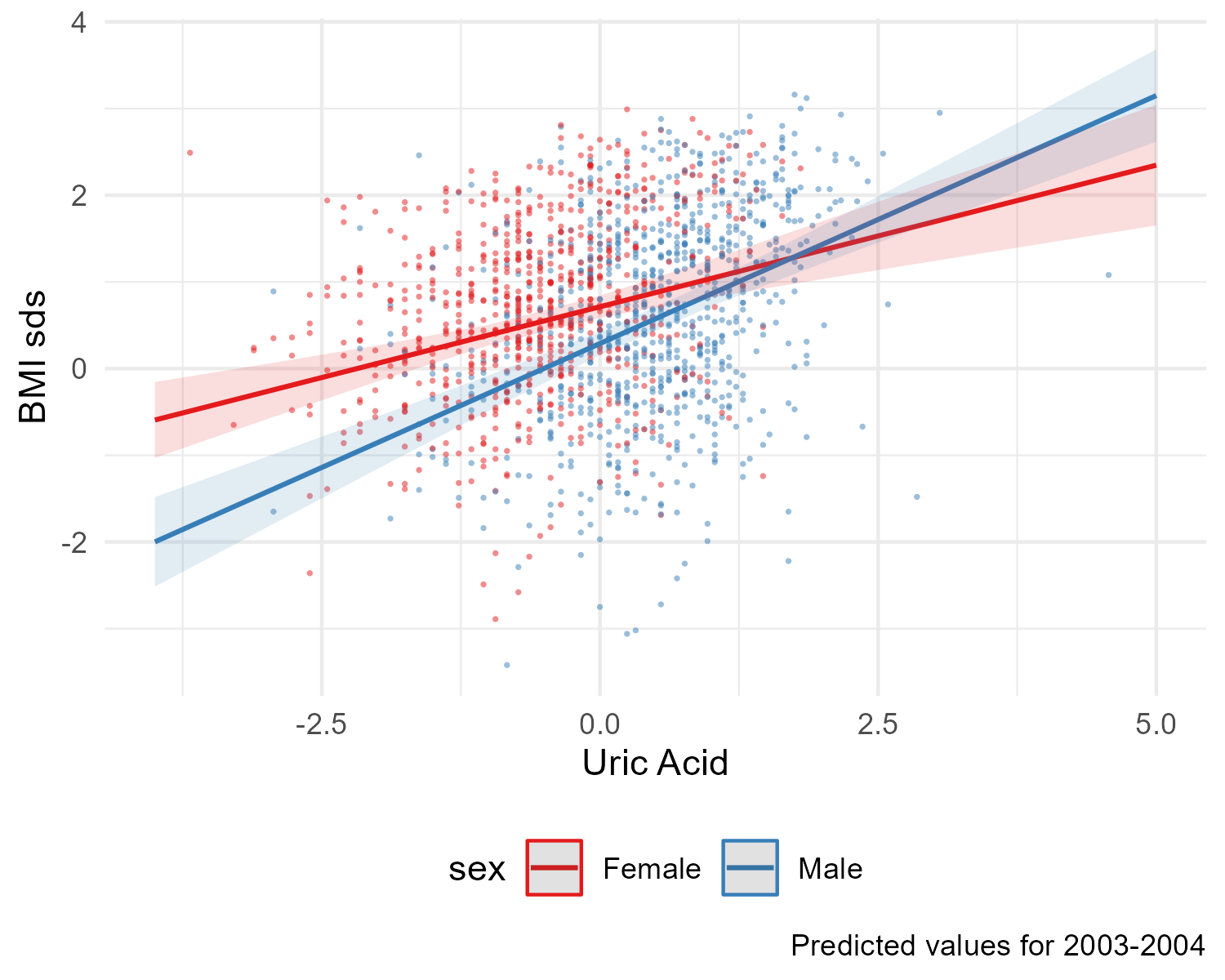

Supplement: Supplementary file 1 — Supplementary Information. [file 41598_2022_12459_MOESM1_ESM.zip › SupplementaryMaterial_DataAnalysisDetails - Copy/figures/figure5.png]

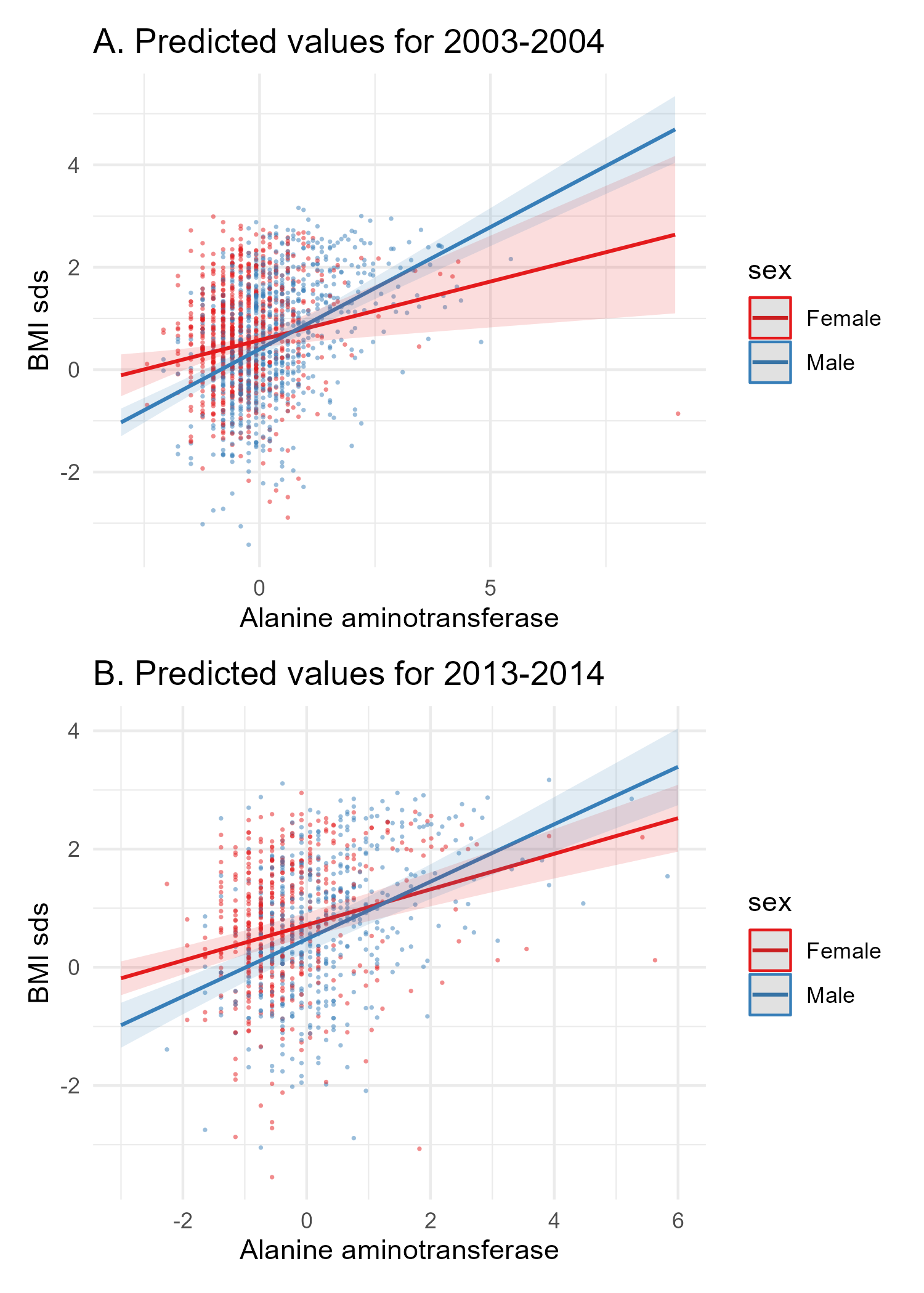

Supplement: Supplementary file 1 — Supplementary Information. [file 41598_2022_12459_MOESM1_ESM.zip › SupplementaryMaterial_DataAnalysisDetails - Copy/figures/figure6.png]
